# Homologous Expression and Characterization of an LPMO from Commensal *Lactiplantibacillus plantarum* WCFS1 and Comparison with Related Enzymes from Chitin-Degrading Bacteria

**DOI:** 10.1021/acsomega.6c02723

**Published:** 2026-06-17

**Authors:** Hanne Berggreen, Tom Z. Emrich-Mills, Geir Mathiesen, Vincent G. H. Eijsink, Zarah Forsberg

**Affiliations:** Faculty of Chemistry, Biotechnology and Food Science, The Norwegian University of Life Sciences (NMBU), Aas 1433, Norway

## Abstract

Lytic polysaccharide monooxygenases (LPMOs) catalyze oxidative cleavage of glycosidic bonds and are important members of Nature’s enzymatic machinery for the deconstruction of recalcitrant polysaccharides. While chitin-active LPMOs are typically found in microorganisms that also produce chitinases, *Lactiplantibacillus plantarum*, a commensal lactic acid bacterium, lacks the chitinolytic machinery yet encodes a single-domain LPMO, hereinafter called *Lp*LPMO10A. Here, we characterize *Lp*LPMO10A produced both homologously and heterologously and demonstrate through structural modeling and sequence comparisons that it groups with canonical chitin-active LPMOs. Biochemical assays show that *Lp*LPMO10A catalyzes oxidative cleavage of chitin, producing C1-oxidized products. Analysis of productive (chitin peroxygenase reaction) and nonproductive (ascorbate peroxidase reaction) H_2_O_2_-consumption, together with additional functional assays, showed that *Lp*LPMO10A is very similar to *Sm*LPMO10A from *Serratia marcescens*, with a known role in chitin degradation. Moreover, *Lp*LPMO10A was more efficient in degrading chitin than *Bl*LPMO10A from *Bacillus licheniformis*, which harbors a chitinolytic gene repertoire including chitinases. For all three LPMOs, these activities showed only minor pH-dependence in the pH 5 – 7 range, meaning that *Lp*LPMO10A does not seem to have a particular potential to act at acidic pH. This lack of pH dependence is shared with fungal LPMOs despite the presence of different titratable groups in the second coordination sphere of the copper. While our results reveal functional variation among chitin-active bacterial LPMOs, for example regarding their ability to withstand oxidative damage, they do not identify distinct functional features that clarify the biological role of *Lp*LPMO10A. Taken together, our findings highlight *Lp*LPMO10A as an evolutionarily intriguing LPMO, namely a chitin-active enzyme retained in a nonchitinolytic bacterium.

## Graphical abstract

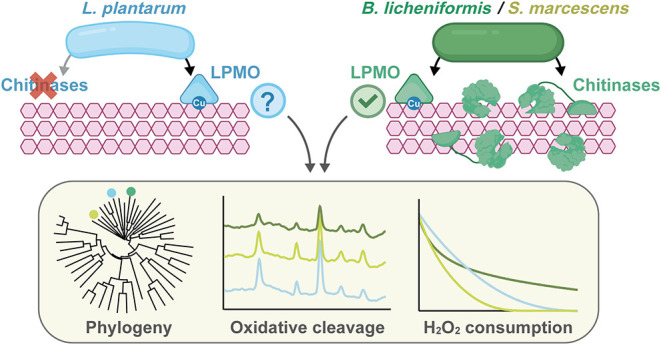

## Introduction

*L. plantarum* is a Gram-positive, facultatively aerobic, rod-shaped lactic acid bacterium (LAB) known for its exceptional metabolic versatility.^1^ Unlike many other LAB, it possesses a relatively large and flexible genome that enables utilization of a wide variety of carbohydrates. *L. plantarum* is often described as a “nomadic” species, reflecting its genomic capacity to metabolize diverse substrates and to thrive in a wide range of ecological niches, including fermented foods, plant materials, and the human and animal gastrointestinal tract.^2,3^ *L. plantarum* grows optimally at pH 6–7 but can tolerate acidic conditions down to pH 4, allowing it to maintain growth despite the accumulation of lactic acid produced during fermentation. A key feature of the metabolic capacity of *L. plantarum* is its broad arsenal of glycoside hydrolases (GHs), which includes members from over a dozen GH families.^4^ These enzymes reflect the known ability of *L. plantarum* to process a wide variety of carbohydrates from dietary plant material, host-derived glycans (such as *N*-glycans and mucins),^5^ and its own peptidoglycan during growth and cell wall remodeling.^6^ The GH profile supports efficient breakdown of oligo- and soluble polysaccharides into fermentable sugars, consistent with a nutritional generalist lifestyle based on scavenging and processing easily accessible glycans rather than deconstructing crystalline polysaccharides such as cellulose and chitin. In this context, the presence of a gene encoding a lytic polysaccharide monooxygenase (LPMO), an enzyme typically associated with oxidative cleavage of recalcitrant substrates, stands out as an intriguing feature of the genome.

LPMOs are copper-dependent redox enzymes that oxidatively cleave glycosidic bonds in crystalline polysaccharides such as chitin, cellulose and other plant cell wall associated glycans.^7–12^ They are classified into eight of the auxiliary activity enzyme families (AA9-AA11 and AA13-AA17) in the CAZy database^13^ and are predominantly found in fungi and bacteria, but also occur in insects, plants, and viruses.^14^ LPMOs are increasingly recognized as multifunctional enzymes, involved not only in biomass degradation but also in pathogenesis, commensalism, and cell wall remodeling.^14–18^ LPMOs share a conserved and distinctive active site featuring a surface-exposed copper ion coordinated by two histidines, often referred to as the histidine brace.^9,19^ The copper is coordinated by three nitrogen atoms; the N-terminal histidine provides both its amino group and imidazole nitrogen and a second histidine contributes another imidazole ligand. Surrounding second-sphere residues modulate the redox properties of the copper and contribute to binding and controlled activation of an oxygen cosubstrate.^20,21^ Mechanistically, LPMOs function primarily as peroxygenases, using hydrogen peroxide (H_2_O_2_) as a cosubstrate^22^ to oxidize the substrate. In addition, LPMOs exhibit oxidase (using O_2_ as cosubstrate) and peroxidase (using H_2_O_2_ as cosubstrate) activities, which are most apparent in the absence of substrate and are often considered as off-pathway reactions.^22–24^ Regardless of whether the enzyme acts as a peroxygenase, peroxidase, or oxidase, its catalytic cycle requires reductive activation of the copper ion and formation of reactive intermediates upon the subsequent reaction with the cosubstrate. Figure 1 provides an overview of LPMO-catalyzed reactions and also lists the assays used to probe these reactions in the present study.

**Figure 1:**
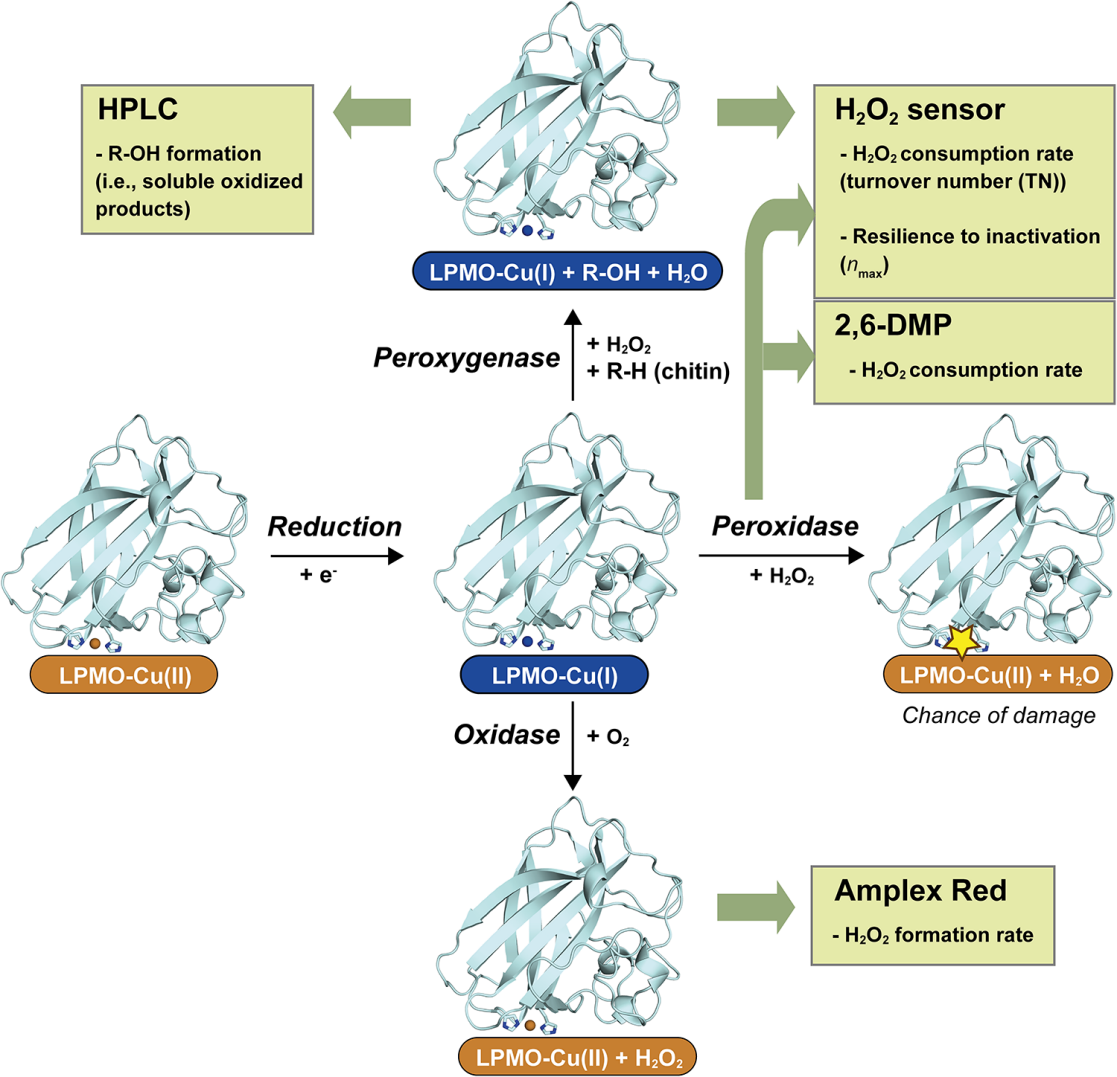
Schematic overview of LPMO reaction pathways examined in this study, indicating the assays used to probe individual steps and the type of information obtained.

Due to the various side reactions and the need for a reductant, kinetic studies of LPMOs are challenging. For example, in reductant-driven LPMO reactions (i.e., reaction without externally added H_2_O_2_), the reaction will be limited by the in situ generation of H_2_O_2_ which is due to the oxidase activity of the LPMO and abiotic oxidation of the reductant. In addition, LPMOs are vulnerable to oxidative damage, particularly under conditions of excess H_2_O_2_ or in the absence of a substrate, i.e., conditions that promote the peroxidase activity. Under such conditions, autocatalytic oxidation of the copper-coordinating histidines can occur, ultimately leading to copper loss from the active site and irreversible enzyme inactivation.^22,25,26^ All these interlinked processes will respond to variations in reaction parameters, such as pH. For example, pH can significantly influence the behavior of reductants that both activate the LPMO and are consumed to generate H_2_O_2_. This makes interpretation of kinetic data challenging, as observed rates reflect a mixture of productive substrate oxidation, side reactions involving free copper and redox cycling, and progressive enzyme inactivation.^27–30^

Phylogenetic analysis shows that the single, one-domain LPMO of *L. plantarum*, *Lp*LPMO10A, clusters with chitin-active AA10 enzymes (Figure 2), suggesting a role in chitin modification. Notably, *L. plantarum* lacks canonical chitinases and is not known to naturally degrade chitin, which leaves the biological role of *Lp*LPMO10A unclear. In this study, we characterized the structural and functional features of *Lp*LPMO10A and compared these with those of two homologous LPMOs from chitinolytic bacteria. Importantly, we expressed and purified *Lp*LPMO10A in both *Escherichia coli* and *L. plantarum*, representing the first reported production of a bacterial LPMO in its native Gram-positive host. This dual expression strategy enabled us to investigate potential host-specific differences in post-translational modifications, enzymatic activity and stability. Additionally, we examined the activity profile across a range of pH values relevant to the acidic growth environment of *L. plantarum* using a newly developed, highly sensitive H_2_O_2_ sensor method that enables precise kinetic analysis of LPMO activity.^30^ Next to providing functional properties of *Lp*LPMO10A, this study, through the comparison with related LPMOs, provides insight into functional variation within the AA10 family.

**Figure 2:**
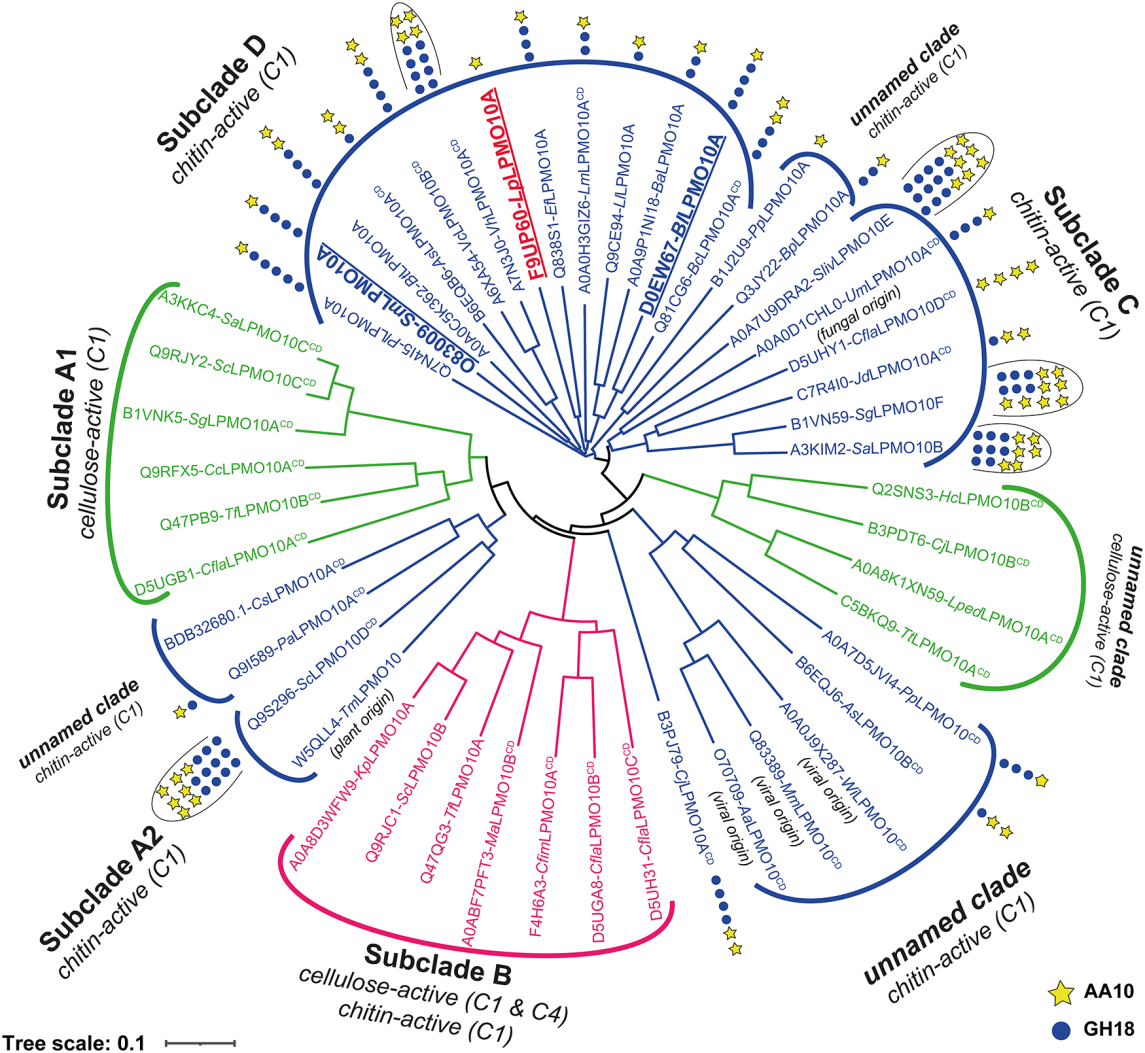
Phylogenetic tree of *Lp*LPMO10A and 47 characterized AA10 LPMOs. The tree was constructed using only the catalytic domains of the enzymes. Functionally or structurally characterized LPMOs are color-coded based on their reported substrate specificity: blue for chitin-active, green for cellulose-active, and pink for enzymes with dual activity. The names of the three enzymes studied in this work, *Bl*LPMO10A, *Lp*LPMO10A, and *Sm*LPMO10A, are enlarged and underlined. Enzymes with additional domains are labeled with “CD” (only catalytic domain used in the phylogenetic analysis), while naturally single-modular enzymes are unlabeled. Additional domains found in AA10-type LPMOs include various carbohydrate-binding modules (CBMs from families 2, 3, 5, 10, 12, and 73), fibronectin type III-like (Fn3) domains, immunoglobulin-like domains, cell wall sorting signals, and glycoside hydrolases from families GH5 (e.g., cellulases/mannanases) and GH18 (e.g., chitinases). For (putatively) chitin-active LPMOs, yellow stars indicate the number of AA10 genes and blue circles the number of GH18 genes found in the genome of the natural producing organism, where such data are available.

## Results and Discussion

### An Orphan LPMO in a Chitinase-Deficient Host

*Lp*LPMO10A consists solely of an AA10 catalytic domain, lacking the carbohydrate-binding modules (CBMs) that are often associated with LPMOs involved in cellulose or chitin degradation.^31^ In addition, several LPMOs reported as virulence factors in humans also contain CBMs, along with additional internal accessory domains thought to contribute to host interactions, pathogenicity, and/or LPMO stability.^15,32–34^ Little is known about *Lp*LPMO10A beyond observations from early studies in which the protein, then considered merely an adherence-associated chitin-binding protein rather than a redox-active enzyme, was identified in the secretome of *L. plantarum* ^35,36^ and shown to bind intestinal mucins and epithelial cells.^37^ To investigate the evolutionary context of *Lp*LPMO10A, we performed phylogenetic analysis of its AA10 domain alongside the AA10 domains of 47 LPMOs that have been characterized functionally, structurally, or both (https://www.cazy.org/AA10.html). The resulting phylogenetic tree revealed that *Lp*LPMO10A clusters within a distinct and well-supported clade (subclade D) composed of C1-oxidizing chitin-active LPMOs, with representatives from both Gram-positive Firmicutes (e.g., *Bacillus*, *Listeria*) and Gram-negative Proteobacteria (e.g., *Vibrio*, *Serratia*) (Figure 2). This clustering indicates that *Lp*LPMO10A likely possesses activity on chitin or chitin-like substrates, despite being encoded by a bacterium that lacks canonical chitinases. Notably, of the 26 organisms with fully annotated genomes and containing putatively chitin-active LPMOs, only Gram-positive *L. plantarum* and Gram-negative *Pseudomonas putida* harbor a single LPMO gene, while lacking chitinases from glycoside hydrolase family 18 (GH18s). In addition, *Cellulomonas flavigena*, primarily associated with cellulose degradation and encoding three cellulose-active LPMOs, also harbors a chitin-clustering LPMO^38^ without accompanying GH18s. The genomes of all other such organisms encode between 1 and 11 chitinases next to one or more LPMOs (Figure 2). This difference in associated chitinolytic machinery suggests that *Lp*LPMO10A may serve alternative functions rather than participating in chitin degradation.

To explore the properties of *Lp*LPMO10A, we selected two previously characterized single-modular AA10 LPMOs for comparison: *Sm*LPMO10A (also known as CBP21 or *Sm*AA10A) from *S. marcescens* ^7,20^ and *Bl*LPMO10A from *B. licheniformis*.^39^ All three enzymes cluster within subclade D and lack additional domains, but only *S. marcescens* and *B. licheniformis* possess established chitinolytic systems.^40–42^ Despite moderate sequence identity (∼50%) and some structural variation (Figure S1), the conservation of key residues involved in substrate-binding^43,44^ and in protective hole-hopping mechanisms^45^ (Figure S1), suggests functional conservation between *Lp*LPMO10A and the other two enzymes.

### Comparison of *Lp*LPMO10A Produced in *L. plantarum* and *E. coli*

Attempts to isolate *Lp*LPMO10A from *L. plantarum* under endogenous constitutive expression resulted in low yields (data not shown) and were therefore not pursued further. To overcome this limitation, we employed the pSIP expression system,^46^ to overexpress *Lp*LPMO10A (*lp_1697*) in *L. plantarum* WCFS1. The full-length *Lp*LPMO10A sequence, including its signal peptide (residue 1–33) was cloned into the expression vector, allowing secretion of the enzyme directly into the culture supernatant (Figure 3A). The *L. plantarum*-expressed LPMO (hereinafter called *Lp*LPMO10A^*Lp*^) was purified from 1 L of culture supernatant. After a 10-fold concentration of the supernatant and a buffer exchange, the enzyme was purified by anion exchange chromatography followed by size exclusion chromatography, yielding 9 mg of purified protein (Figure 3B).

**Figure 3:**
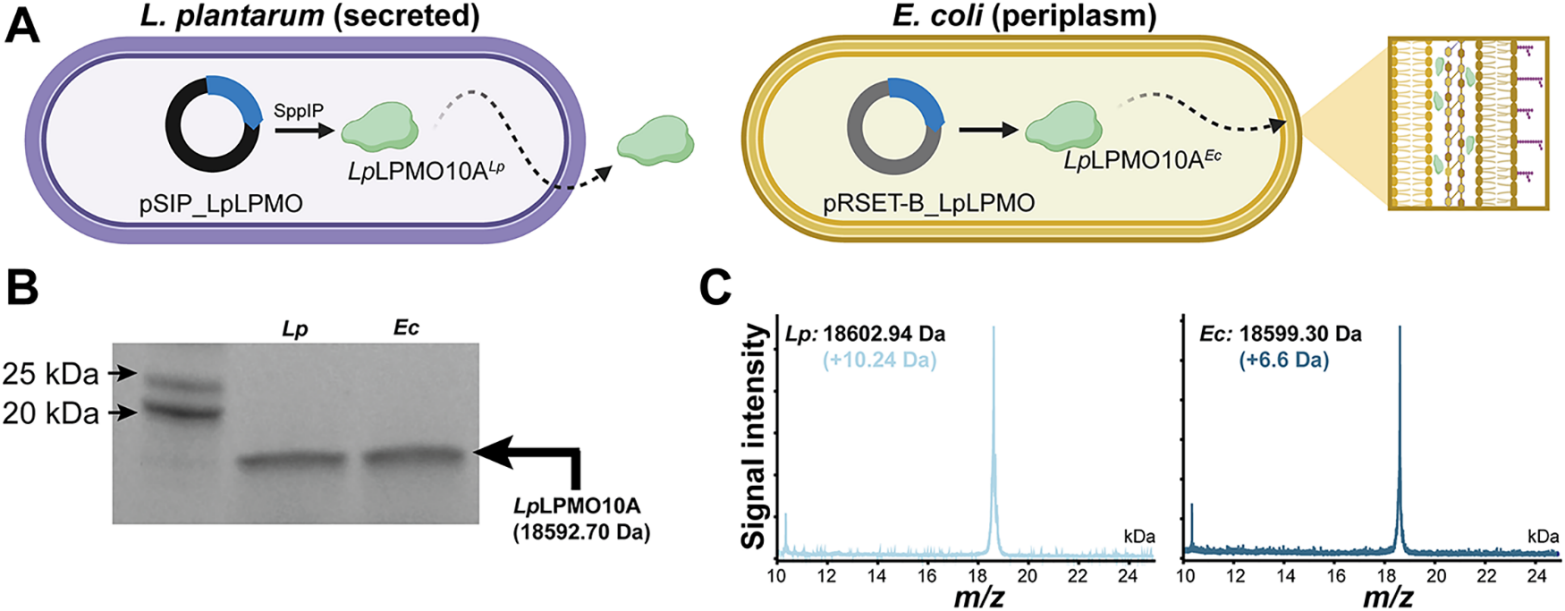
Comparison of *Lp*LPMO10A produced in different bacterial expression systems. Panel (A) shows a schematic overview of the expression systems used. In *L. plantarum* WCFS1, the pSIP vector^48^ carries the *Lp*LPMO10A gene, including its native signal peptide, leading to secretion of the enzyme directly into the culture supernatant upon induction of gene expression with the inducer peptide SppIP. In *E. coli* BL21­(DE3) Star, the LPMO gene is inserted in the pRSET-B vector with the native signal peptide being replaced with the signal peptide of *Sm*LPMO10A, known to promote efficient translocation to the periplasm.^49^ Due to promoter leakiness, induction was not required. Panel (B) shows SDS-PAGE analysis of purified, copper-saturated *Lp*LPMO10A expressed in *L. plantarum* (*Lp*) or *E. coli* (*Ec*). The predicted molecular mass of *Lp*LPMO10A without its signal peptide is 18592.7 Da. Panel (C) shows whole-protein MALDI-ToF MS spectra of *Lp*LPMO10A^*Lp*^ and *Lp*LPMO10A^*Ec*^, with deviations from the predicted mass shown in parentheses.

To determine whether expression in the native host introduced post-translational modifications (PTMs), we compared *Lp*LPMO10A^*Lp*^ produced in *L. plantarum* WCFS1, which is known to secrete glycosylated proteins,^47^ with the enzyme expressed in the periplasm of *E. coli* BL21­(DE3) Star using the pRSETB vector system (hereinafter referred to as *Lp*LPMO10A^*Ec*^; typical yield of 10 mg purified protein per liter of culture). SDS-PAGE analysis of the enzymes produced in the two hosts showed no apparent differences in electrophoretic mobility (Figure 3B), indicating the absence of substantial PTMs affecting size or charge. Further investigation, using whole-protein MALDI-ToF analysis, revealed no evidence of PTMs such as phosphorylation, lactylation, acetylation, or glycosylation (Figure 3C). However, the broad peaks in the mass spectra preclude a definitive exclusion of subtle modifications. Thus, both enzyme variants were included in subsequent experiments to enable robust comparative analysis.

Oxidative activity toward both α- and β-chitin was confirmed for all LPMOs (i.e., *Lp*LPMO10A^*Ec*^, *Lp*LPMO10A^*Lp*^, *Sm*LPMO10A and *Bl*LPMO10A) by quantification of oxidized products after 24 h of incubation (Figure S2). All enzymes showed significantly higher product yields in reactions with β-chitin compared to α-chitin, and β-chitin was therefore used as substrate in subsequent experiments. Notably, *Sm*LPMO10A was the most active enzyme overall, retaining considerable activity on α-chitin, whereas the other enzymes were minimally active on this substrate. In this analysis, soluble oxidized products were converted to a mixture of GlcNAc monomers and oxidized GlcNAc dimers; the fact that reactions with β-chitin showed very similar ratios of these two products for all enzymes (Figure S2) shows that they degrade chitin in a similar manner. Product formation by *Lp*LPMO10A^*Lp*^ and *Lp*LPMO10A^*Ec*^ was further confirmed by hydrophilic interaction chromatography and MALDI-ToF MS (Figure S3). This latter analysis also revealed identical product profiles for *Lp*LPMO10A^*Lp*^, *Lp*LPMO10A^*Ec*^, and the two other LPMOs (*Bl*LPMO10A and *Sm*LPMO10A).

### Effects of pH on Protein Integrity

The microenvironment surrounding lactic acid bacteria becomes increasingly acidic due to the secretion of lactic acid during metabolic activity. Since the LPMO is also secreted, a tolerance to acidic conditions could be advantageous for maintaining enzymatic activity and stability. Therefore, most experiments described below were conducted at pH 5.0, representing the acidic conditions that *L. plantarum* may encounter in its surroundings. In addition, we did experiments using pH 6–8, a pH range commonly used in studies of bacterial LPMOs.

Protein integrity of LPMOs under different pH conditions can be assessed by determining the apparent melting temperature (T_*m*,app_). To examine the T_*m*,app_ of *Lp*LPMO10A across a broad pH range from low to neutral pH (pH 3–7), we employed a universal buffer system (10 mM K_3_BO_3_, CH_3_COONa, Na_2_HPO_4_) to minimize buffer-specific effects (Table 1). Comparison of the T_*m*,app_ of copper-saturated *Lp*LPMO10A^*Lp*^ and *Lp*LPMO10A^*Ec*^, revealed only minor differences between the two variants across the tested pH range. The highest T_*m*,app_ was observed at pH 5.0 (73.1 ± 0.0 and 72.6 ± 0.4, respectively) and the lowest at pH 4.0 (69.6 ± 0.2 and 68.2 ± 0.6, respectively). At pH 3.0, no clear melting transition was observed, consistent with pH-induced unfolding rather than temperature-driven denaturation. Of note, pH 3.0 lies well below the normal growth range of *L. plantarum* WCFS1.^50^ Overall, these results indicate that pH exerts only a modest influence on the folding stability of *Lp*LPMO10A between pH 4 and 7, with maximal stability occurring at pH 5.0.

**Table 1:** Apparent Melting Temperatures Measured by DSF^a^,^b^

*T*_*m*(app)_ (*°C*)	*Lp*LPMO10A^*Lp*^	*Lp*LPMO10A^*Ec*^	*Sm*LPMO10A	*Bl*LPMO10A
universal, pH 3.0	n.d.	n.d.	-	-
universal, pH 4.0	69.6 ± 0.2	68.2 ± 0.6	-	-
universal, pH 5.0	73.1 ± 0.0	72.6 ± 0.4	-	-
universal, pH 6.0	72.3 ± 0.0	71.6 ± 0.2	-	-
universal, pH 7.0	70.4 ± 0.2	69.7 ± 0.2	-	-
NaOAc, pH 5.0	73.5 ± 0.2	73.4 ± 0.2	74.1 ± 0.0	70.9 ± 0.0
Tris-HCl, pH 8.0	69.2 ± 0.2	69.6 ± 0.2	69.3 ± 0.0	74.4 ± 0.4

a Differential Scanning Fluorimetry

b All experiments were performed in triplicate. n.d., not possible to determine (see main text); -, not determined

For comparison, the apparent melting temperatures of *Lp*LPMO10A, *Sm*LPMO10A and *Bl*LPMO10A were determined at low (sodium acetate, pH 5.0) and high (Tris-HCl, pH 8.0) pH (Table 1). Overall, the various T_*m*,app_ values were similar. Like *Lp*LPMO10A^*Ec*^ and *Lp*LPMO10A^*Lp*^, *Sm*LPMO10A showed a slightly higher T_*m*,app_ (74.1 ± 0.0 °C) at pH 5.0 compared to pH 8.0 (69.3° ± 0.0 C). On the other hand, *Bl*LPMO10A had a lower T_*m*,app_ at pH 5.0 (70.9 ± 0.0 °C) compared to pH 8.0 (74.4 ± 0.4 °C). It is worth noting that *Lp*LPMO10A, *Sm*LPMO10A and *Bl*LPMO10A show similar stabilities despite a conspicuous difference in the number of disulfide bridges (zero, two and one, respectively; Figure S1B).^51^

### Effect of pH on Binding to Chitin

Chitin-binding was assessed using reducing conditions, since LPMOs are known to bind more strongly to their substrates when reduced.^52^ At pH 5.0 (NaOAc buffer), all LPMOs exhibited similar binding, with approximately 55–65% of the protein being associated with the substrate after 5 min, at which point the level of binding became stable. At pH 8.0 (Tris-HCl buffer), *Lp*LPMO10A showed lower binding (∼45–50%) compared to *Sm*LPMO10A and *Bl*LPMO10A, both of which showed ∼65–70% binding (Figure 4). While *Sm*LPMO10A and *Bl*LPMO10A seem to bind slightly more strongly than *Lp*LPMO10A at pH 5.0, these differences were within experimental variation. At pH 8.0, however, the difference is pronounced: *Lp*LPMO10A exhibited reduced binding, whereas *Sm*LPMO10A and *Bl*LPMO10A showed similar or slightly increased binding compared to pH 5.0. These results suggest that *Lp*LPMO10A is adapted to binding its substrate in acidic conditions.

**Figure 4:**
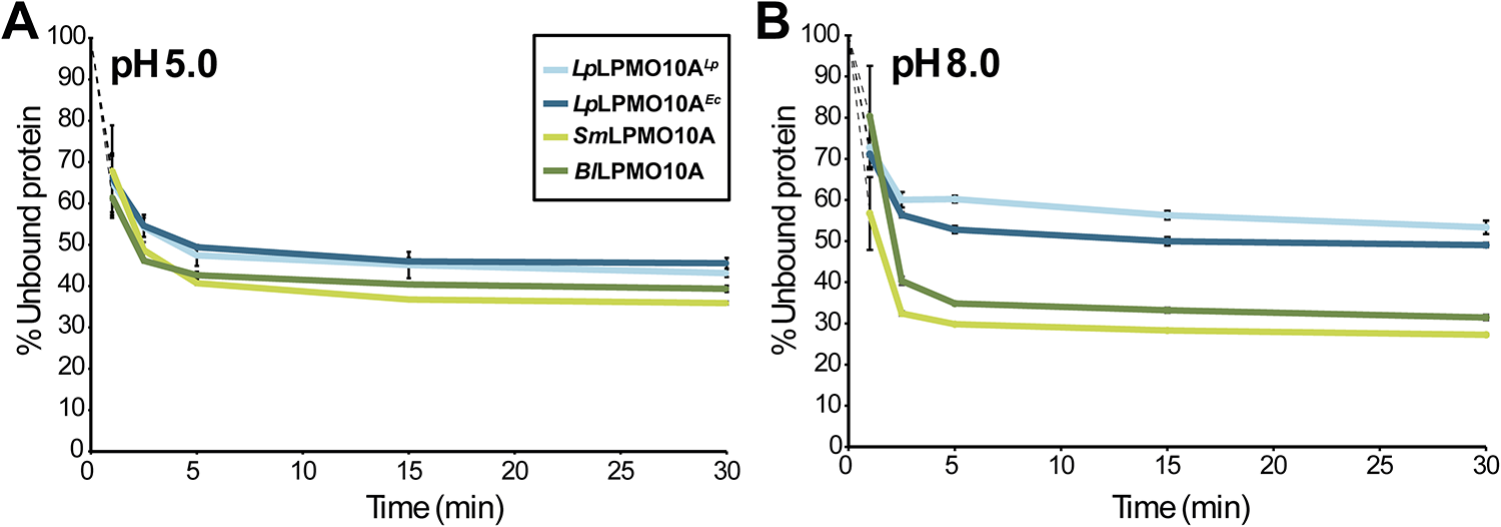
LPMO binding to β-chitin at pH 5.0 and 8.0. Binding of *Lp*LPMO10A^*Lp*^, *Lp*LPMO10A^*Ec*^, *Sm*LPMO10A and *Bl*LPMO10A to β-chitin was measured in the presence of ascorbic acid in (A) 50 mM NaOAc (pH 5.0) or (B) 50 mM Tris-HCl (pH 8.0). Reactions containing 10 g/L β-chitin, 2 μM copper-loaded LPMO and 1 mM ascorbic acid were incubated at 22 °C, 800 rpm, and sampled over time. Unbound enzyme was separated from the insoluble chitin fraction by filtration and quantified using a modified Bradford assay.

### Comparative Analysis of Off-Pathway Peroxidase and Oxidase Activities

The peroxidase activity of the LPMOs at pH 5.0 was first assessed using the 2,6-dimethoxyphenol (2,6-DMP) assay described by Breslmayr et al.^53^ In this assay, 2,6-DMP reduces the LPMO, and the oxidized 2,6-DMP spontaneously dimerizes to form hydrocoerolignone. In a second step, the reduced LPMO is reoxidized by H_2_O_2_, and the resulting oxidized LPMO reacts with hydrocoerulignone to form the yellow product coerulignone, which can be monitored spectrophotometrically at 469 nm. Clear differences were observed: *Bl*LPMO10A displayed the highest apparent peroxidase activity, with an initial rate of 0.070 ΔA469/min, followed by *Sm*LPMO10A with an intermediate rate of 0.047 ΔA469/min, and the two preparations of *Lp*LPMO10A showing an average apparent initial rate of 0.025 ΔA469/min (Figure 5A). A limitation of the 2,6-DMP assay is that the catalytic rate may be restricted by the reduction of the LPMO by 2,6-DMP. Depending on the redox potential of the LPMO, this can result in an apparent peroxidase activity that underestimates the true peroxidase rate. Therefore, peroxidase activity was also evaluated using the recently developed electrochemical H_2_O_2_ sensor (Figure 5B) described by Schwaiger et al.,^30^ which enables accurate measurement of H_2_O_2_ consumption by LPMOs in the presence of millimolar concentrations of ascorbic acid as reductant. The results confirmed that *Lp*LPMO10A is the slowest peroxidase of the three enzymes tested (TN 0.21 ± 0.05 s^–1^), whereas *Sm*LPMO10A (TN 0.28 ± 0.008 s^–1^) and particularly *Bl*LPMO10A (TN 1.22 ± 0.12 s^–1^) showed higher activities (Figure 5C).

**Figure 5:**
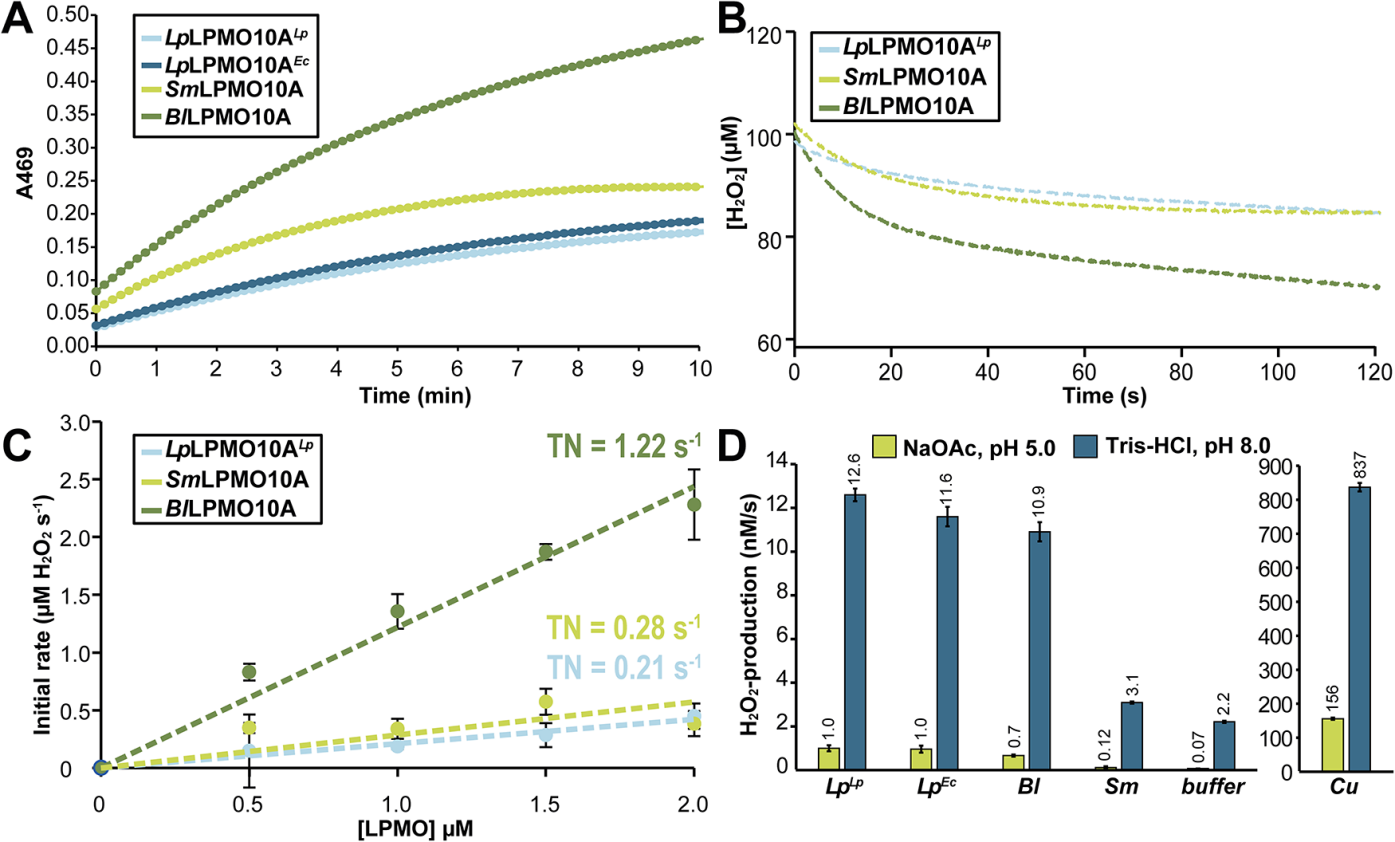
Peroxidase and oxidase activity of LPMOs. Panel (A) shows the apparent peroxidase activity of the LPMOs based on LPMO-catalyzed oxidation of 2,6-DMP. Reactions were carried out in a Varioskan LUX plate reader set to 30 °C and contained 2 μM LPMO, 2 mM 2,6-DMP, and 200 μM H_2_O_2_ in 50 mM NaOAc buffer (pH 5.0). For clarity, one representative curve per enzyme is shown; each represents the average of three technical replicates (*n* = 3), which showed negligible variation. Panel (B) shows raw data for measurement of the peroxidase activity through electrochemical real-time monitoring of H_2_O_2_ consumption for 1 μM LPMO. Panel (C) shows the initial rates of peroxidase activity plotted against LPMO concentration, derived from measurements performed as in panel (B) but at multiple LPMO concentrations. The slope of the curve defines the turnover number (TN, s^–1^), representing the number of H_2_O_2_ molecules consumed per enzyme under the following conditions: 0.5–2 μM Cu-loaded LPMO, 1 mM ascorbic acid, 100 μM H_2_O_2_, 50 mM NaOAc pH 5.0, and 100 mM KCl, at 37 °C. Initial rate values were corrected for the rates found in control experiments without LPMO. Panel (D) shows the apparent oxidase activity expressed as the rate of H_2_O_2_ production (nM/s), measured using the Amplex red/HRP assay. Reactions contained 3 μM LPMO or 3 μM Cu­(II)­SO_4_, 5 U/mL HRP, 100 μM Amplex red, 1 mM AscA in either 25 mM sodium acetate buffer (pH 5.0), or Tris-HCl (pH 8.0) at 30 °C (*n* = 3).

The oxidase activity of the various LPMOs, reflected in the production of H_2_O_2_, was measured in the absence of substrate using the Amplex Red/horseradish peroxidase (HRP) assay.^54^ Under these conditions, all LPMOs displayed detectable oxidase activity, although activities were markedly lower at pH 5.0 than at pH 8.0 (Figure 5D). The strongly reduced activity observed at acidic pH is consistent with ascorbic acid becoming a rate-limiting reductant under these conditions,^27^ as protonation at low pH substantially restricts its electron-donating capacity.^55^ The LPMOs (at 3 μM) exhibited oxidase rates in the range of ∼0.12–1 nM s^–1^ at pH 5.0 and ∼11–13 nM s^–1^ at pH 8, with the exception of *Sm*LPMO10A that showed a lower rate of 3.1 nM s^–1^ at pH 8.0, only marginally above the background signal detected in the buffer control (2.2 nM s^–1^). For comparison, free copper (3 μM) showed oxidase rates of 156 ± 3 nM s^–1^ at pH 5.0 and 837 ± 12 nM s^–1^ at pH 8.0, highlighting the substantially lower activities observed for the enzymes. The low oxidase activity of *Sm*LPMO10A is consistent with previously reported observations for this enzyme.^56^ While the oxidase activities of *Lp*LPMO10A and *Bl*LPMO10A were higher than that of *Sm*LPMO10A, these activities remain low compared with LPMOs from other families (e.g., AA9s and AA11s), which can exhibit oxidase rates that are 1 order of magnitude higher.^21,56^

### Comparative Analysis of the On-Pathway Peroxygenase Activity

We next examined the peroxygenase activity of the LPMOs on chitin. In reductant-driven reactions, without an external H_2_O_2_ source, product formation over 60 min was similar and low for all LPMOs with *Sm*LPMO10A, which has low oxidase activity, being somewhat slower than the other two enzymes (Figure S4). This does not necessarily mean that the enzymes have similar activities because the rate of these reductant-driven reactions is limited by the in situ generation of H_2_O_2_. Given the low oxidase rates of all three enzymes (Figure 5D), in situ H_2_O_2_ generation is largely driven by ascorbate-dependent reduction of dissolved O_2_,^22,28^ resulting in similar apparent peroxygenase rates.

To overcome this limitation and directly compare enzyme activity and H_2_O_2_ tolerance, we supplied the reactions with defined concentrations of exogenous H_2_O_2_ (up to 200 μM) at pH 5.0, and quantified both chitin degradation products (Figure 6A–C) and H_2_O_2_ consumption (Figure 6D). Since the two *Lp*LPMO10A variants showed no differences in previous experiments, nor in the experiment depicted in Figure 6A, and shown for both variants in Figure S4, all subsequent experiments were carried out with the *L. plantarum* expressed variant (*Lp*LPMO10A^*Lp*^).

**Figure 6:**
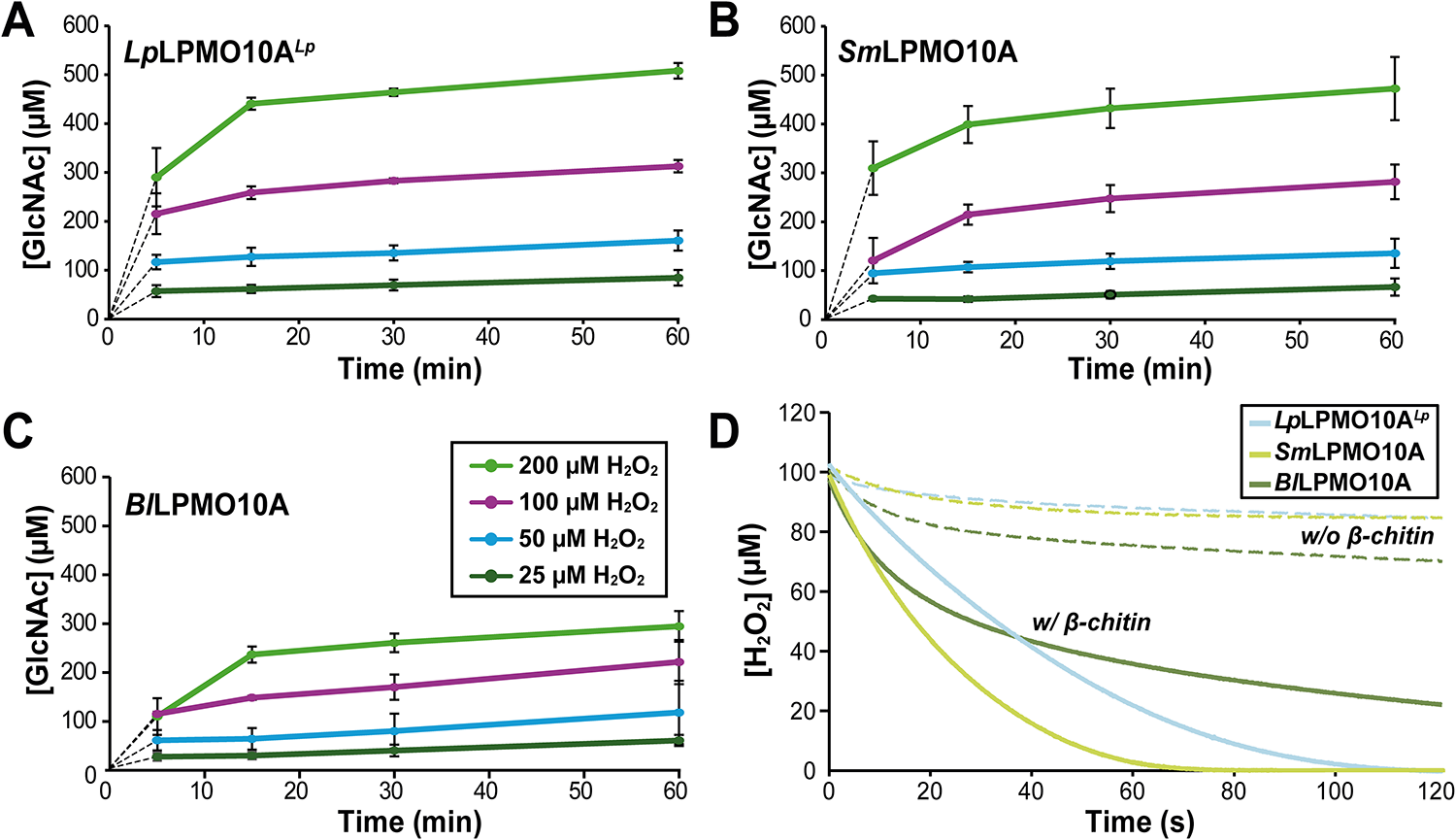
The effect of H_2_O_2_ on LPMO activity at pH 5.0. The panels show time-courses for chitin degradation by 1 μM *Lp*LPMO10A^*Lp*^ (A), *Sm*LPMO10A (B) or *Bl*LPMO10A (C) in the presence of different initial concentrations of exogenous H_2_O_2_ (25–200 μM) and ascorbic acid (1 mM). Progress curves for *Lp*LPMO10A^*Ec*^ were essentially identical to those obtained with *Lp*LPMO10A^*Lp*^, as shown in Figure S4. Product formation over time in the absence of supplemented H_2_O_2_, which is much slower, is also shown in Figure S4. Reactions containing 10 g/L β-chitin in 25 mM NaOAc (pH 5.0) were initiated by the addition of ascorbic acid and incubated at 37 °C with shaking (800 rpm). Samples were taken at specified time points and filtered. Soluble oxidized chito-oligosaccharides were converted to GlcNAc and GlcNAcGlcNAc1A by treatment with *Sm*CHB followed by quantification of GlcNAc by UHPLC. Error bars show ± s.d. (*n* = 3). Panel (D) shows H_2_O_2_ consumption, monitored with an electrochemical H_2_O_2_ sensor, by the three LPMOs in the presence (peroxygenase reaction; solid lines) or absence (peroxidase reaction; dashed lines) of 10 g/L β-chitin, where the dashed lines are identical to those shown in Figure 5B. Curves are means of 3 technical replicates, error bars are not shown for clarity. The initial rates of peroxygenase activity were estimated by subtracting the initial rates of reactions without β-chitin and rates from the background reactions with chitin but without LPMO (not shown) from the observed rates with β-chitin,^30^ yielding rates of 1.47 ± 0.15 s^–1^ for *Lp*LPMO10A, 2.97 ± 0.16 s^–1^ for *Sm*LPMO10A and 2.40 ± 0.13 s^–1^ for *Bl*LPMO10A (means ± s.d.; *n* = 3 technical replicates). The reactions contained 1 μM LPMO, 1 mM ascorbic acid, 50 mM NaOAc (pH 5.0), 100 mM KCl and 100 μM H_2_O_2_.

The product formation curves for reactions with added H_2_O_2_ (Figure 6A–C) show a very fast initial phase in which all added H_2_O_2_ is consumed, followed by a slow phase in which the peroxygenase reaction becomes reductant-driven and is limited by (slow) in situ generation of H_2_O_2_. The progress curves for *Lp*LPMO10A and *Sm*LPMO10A are similar and do not show obvious signs of enzyme inactivation, whereas progress curves for *Bl*LPMO10A show lower product levels, indicative of enzyme inactivation. In principle, variation in product levels could also reflect variations in product profiles (i.e., the degree of polymerization of solubilized oxidized chito-oligosaccharides) since product quantification is based on detection of *N*-acetylglucosamine, however, as discussed above, the LPMOs studied here showed very similar product patterns (Figures S2 and S3).

To obtain more precise insight into these differences and to obtain catalytic rates for the peroxygenase activity, reactions with chitin were followed by measuring H_2_O_2_ consumption in real time using the electrochemical sensor (Figure 6D). The results for reactions, run with a starting H_2_O_2_ concentration of 100 μM, show that *Sm*LPMO10A and *Bl*LPMO10A react faster (2.97 ± 0.16 s^–1^ and 2.40 ± 0.13 s^–1^, respectively) compared to *Lp*LPMO10A (1.47 ± 0.15 s^–1^). These initial rates compare well with previously published rates for AA10-type LPMOs, using a very different^57^ as well as a similar^29^ experimental approach.

While the progress curves for *Sm*LPMO10A and *Lp*LPMO10A have similar shapes, the shape of the progress curve for *Bl*LPMO10A is conspicuously different (Figure 6D). The shape of this latter curve suggests that *Bl*LPMO10A is more rapidly inactivated, as also suggested by the product formation curves of Figure 6A–C.

### A Closer Look at H_2_O_2_ Resilience

To assess enzyme inactivation in the peroxygenase reaction, a second addition of 100 μM H_2_O_2_ was made to the reactions after the first dose had been consumed. The resulting H_2_O_2_ depletion curves (Figure 7A–C) show that after consuming the first portion of H_2_O_2_ *Sm*LPMO10A and *Lp*LPMO10A retain ∼75% and ∼90% of their initial activity, respectively. In contrast, *Bl*LPMO10A loses over 90% of its activity, confirming the lower stability of this enzyme under turnover conditions. To investigate possible causes of this difference in stability, we assessed the extent to which the off-pathway peroxidase reaction damages the enzymes. Specifically, we measured the amount of H_2_O_2_ depleted before the peroxidase rate reached the background rate, which indicates that the enzyme is fully inactivated. By performing these measurements at different enzyme concentrations, we determined *n*_max_,^24^ i.e., the average number of peroxidase reactions catalyzed by one LPMO molecule before inactivation, for each LPMO (Figure 7D). *Lp*LPMO10A showed a turnover number (TN; Figure 5C) of 0.21 s^–1^ and a *n*_max_ value of 7.3 (Figure 7D). By comparison, *Sm*LPMO10A displayed a TN of 0.28 s^–1^ and a *n*_max_ of 10.5, agreeing well with previous reports.^24^ *Bl*LPMO10A was the fastest and most resilient peroxidase (TN 1.22 s^–1^; *n*_max_ 16.6). Interestingly, this comparative analysis reveals a compensatory mechanism that has previously been observed when comparing AA9 and AA10 LPMOs^24^: the LPMO that is most likely to be damaged by the peroxidase reaction, *Lp*LPMO10A, also shows the lowest tendency to engage in this reaction. Comparison of the *n*_max_/TN values, representing the lifetime of the LPMO, suggests that, in the absence of substrate, both *Lp*LPMO10A (34.8 s) and *Sm*LPMO10A (37.5 s) have a longer lifetime than *Bl*LPMO10A (13.6 s).

**Figure 7:**
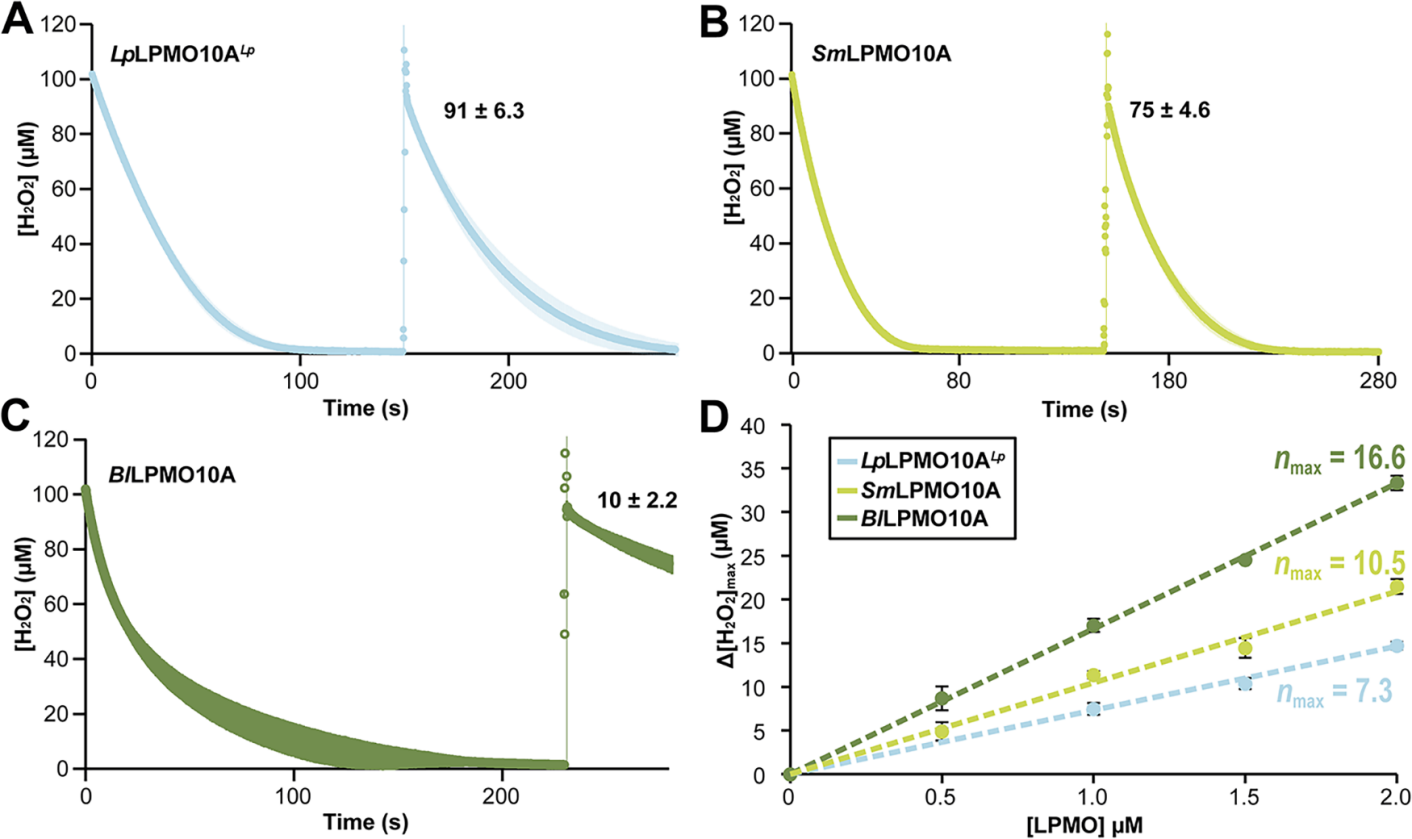
Residual peroxygenase activity and peroxidase resilience. Panel (A–C) shows real-time H_2_O_2_ consumption in reactions containing 1 μM LPMO, 10 g/L β-chitin, 1 mM ascorbic acid, 50 mM NaOAc (pH 5.0), 100 mM KCl, and 100 μM initial H_2_O_2_. LPMO was preincubated with β-chitin and H_2_O_2_, then ascorbic acid was added at *t* = 0 to trigger the reaction. A second addition of 100 μM H_2_O_2_ was made following complete consumption of the first 100 μM of H_2_O_2_. The initial rate of the second depletion was divided by that of the first depletion to estimate percentage residual activity, which is indicated in the panels. Panel (D) shows the total amount of H_2_O_2_ turned over before enzyme inactivation as a function of LPMO concentration in the absence of substrate. The slope defines *n*_max_, the average number of peroxidase reactions a single LPMO can perform before inactivation under these conditions (0.5–2 μM Cu-loaded LPMO, 1 mM ascorbic acid, 100 μM H_2_O_2_, 50 mM NaOAc pH 5.0, and 100 mM KCl, at 37 °C).

Based on the above results, the low stability of *Bl*LPMO10A during chitin turnover is remarkable. Weaker substrate binding, which would lead to a higher chance of peroxidase reactions taking place, could be an explanation, but this is not supported by the binding studies depicted in Figure 4. The higher peroxidase rate of *Bl*LPMO10A cannot explain lower stability either, since this high rate comes with the highest *n*_max_ of the three enzymes (Figure 7D). As alluded to above, *Bl*LPMO10A has the lowest “off-pathway” lifetime of the three LPMOs, but the differences in lifetime are not sufficient to explain the drastically lower stability of the *Bacillus* enzyme during chitin turnover. Thus, it seems that the lower stability of *Bl*LPMO10A during turnover somehow is related to the actual reaction with β-chitin. Although highly speculative, one could envisage that *Bl*LPMO10A binds the β-chitin in a slightly different manner than the other two enzymes, perhaps leading to less precise confinement of the radicals formed during catalysis and a bigger chance of such radicals damaging the enzyme instead of oxidizing the substrate.

A recent study using ancestral sequence reconstruction^58^ revealed features of the core of *Sm*LPMO10A that are important for redox stability because they affect the peroxidase activity and modulate a hole-hopping pathway that helps protect the active site from radicals going astray.^59^ In particular, this study demonstrated the importance of an aspartate residue modulating the orientation of a buried tryptophan and also pointed at a possible role of an aromatic residue at the hole hopping exit point.^58^ Interestingly, while these residues (and the positioning of the tryptophan) are shared by *Sm*LPMO10A and *Lp*LPMO10A, they are lacking in *Bl*LPMO10A (Figure 8). Based on the recent study by Ayuso-Fernandez et al.,^58^ one would thus expect *Bl*LPMO10A to show lower redox stability and a higher peroxidase activity than the other two LPMOs, which is in accordance with the results presented above.

**Figure 8:**
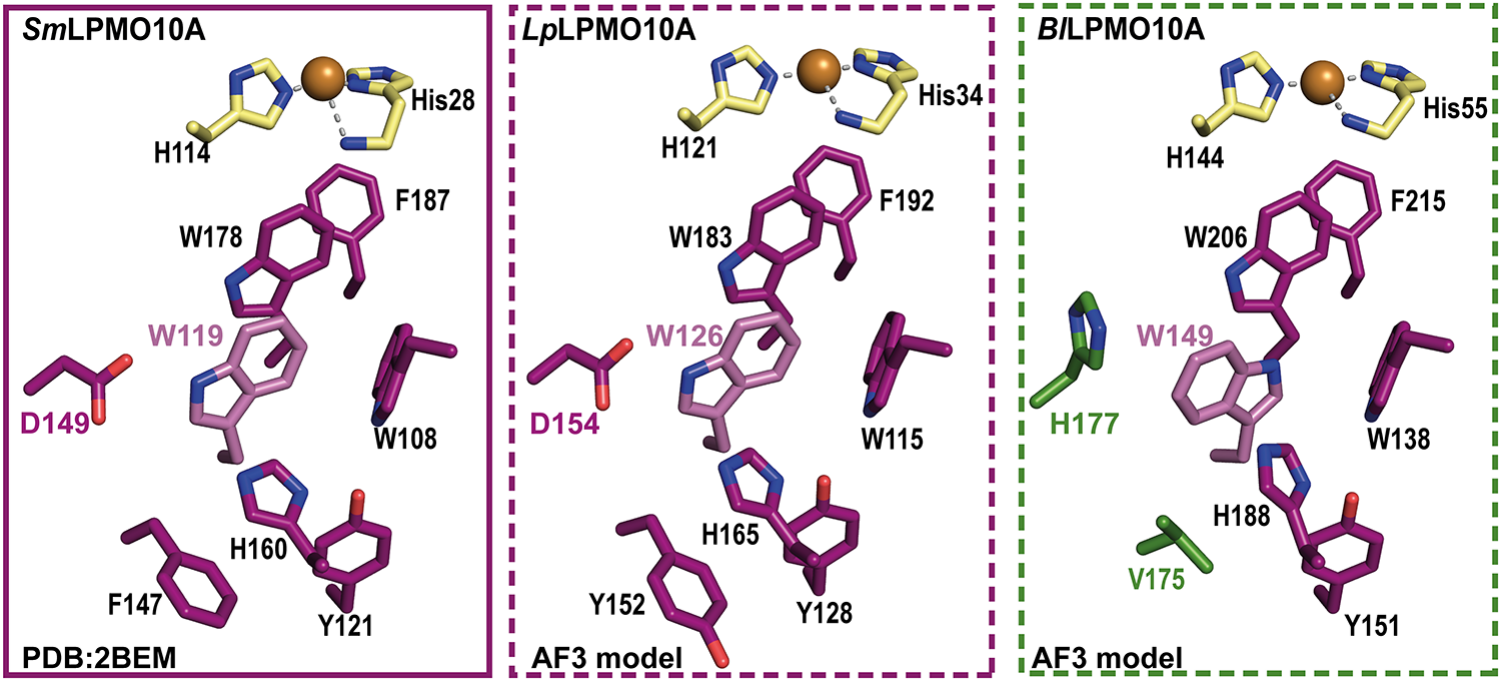
Potential hole hopping pathways. The occurrence of hole hopping and the involvement of the shown residues has been experimentally demonstrated for *Sm*LPMO10A.^45,58^ It has also been shown for *Sm*LPMO10A that D149, and to a lesser extent F147, contribute to redox robustness by modulating the orientation (W119) and/or chemical environment (H160) of residues in the hole hopping pathway.^58^ In the absence of D149, W119 adopts another conformation, as is visible in *Bl*LPMO10A (W149). Note that D149 and an aromatic residue at position 147 in *Sm*LPMO10A are conserved in *Lp*LPMO10A, but not in *Bl*LPMO10A.

### Further Insights into the pH Dependence of *Lp*LPMO10A Activity

Studying the pH dependence of LPMO activity is complicated because pH affects the redox properties of the reductant^27^ and affects each of the on- and off-pathway reactions catalyzed by the LPMO.^60^ Despite the difficulty of disentangling *true* pH effects on LPMO reactions, such effects have been revealed, for AA9-type LPMOs only, in recent kinetic studies by Kuusk et al.^60^ and Schwaiger et al.^30^ The latter study employed the electrochemical H_2_O_2_-sensor to directly monitor H_2_O_2_ consumption, which allows determination of the pH-activity profile without relying solely on reductant-dependent readouts. While this approach still requires careful consideration of how pH may influence the reductants themselves,^30^ it offers a sound method for evaluating LPMO peroxygenase activity across a physiologically and mechanistically relevant pH range.

For all three AA10 LPMOs examined here, we observed a gradual increase in the peroxidase rate with increasing pH that was somewhat more pronounced for *Bl*LPMO10A (Figure 9). Studying fungal *Tr*LPMO9A and measuring ascorbic acid depletion to monitor the peroxidase reaction, Kuusk et al. found a similar gradual increase in the pH 5.0–7.0 range. As discussed extensively by Kuusk et al.,^60^ LPMO reduction is likely to be rate-limiting under these conditions, and this reduction tends to become gradually easier at increased pH, as shown by single turnover reduction experiments with fungal *Nc*LPMO9C.^30^ All in all, Figure 9 shows that, in terms of the pH dependence of the peroxidase reaction, AA9 and AA10 LPMOs are similar, despite having considerably different second copper coordination spheres. Furthermore, the results emphasize that *Lp*LPMO10A is very similar to *Sm*LPMO10A.

**Figure 9:**
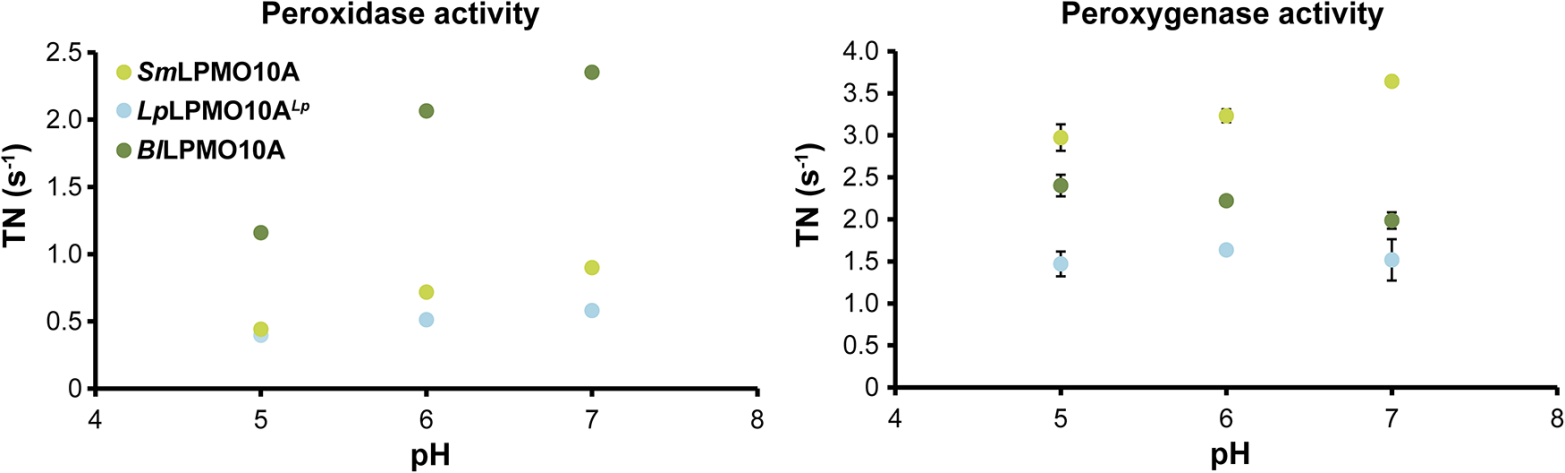
pH-dependent peroxidase and peroxygenase activity of three AA10 LPMOs. Peroxidase and peroxygenase activities of *Bl*LPMO10A, *Lp*LPMO10A^*Lp*^, and *Sm*LPMO10A were estimated by monitoring H_2_O_2_ consumption rates at pH 5.0, 6.0, and 7.0. Peroxidase activity data derives from the initial rate of H_2_O_2_ depletion in the absence of chitin, while peroxygenase data derives from the initial rate of H_2_O_2_ depletion in the presence of 10 g/L β-chitin minus the peroxidase activity (see methods section for details). The background H_2_O_2_ depletion in experiments with chitin but without LPMO was subtracted from all initial rates. The uncorrected H_2_O_2_ consumption data for all individual reactions are shown in Figure S5, which also shows the pH profile for the peroxygenase rates corrected only for the no-enzyme background control, but not for the peroxidase reaction (see text for details). Lastly, rate values were divided by enzyme concentration to give the turnover number (TN, s^–1^). Experiments were conducted in 50 mM NaOAc, pH 5.0, or 50 mM MES buffer, at pH 6.0 or 7.0, at 37 °C using 1 μM LPMO and 100 μM H_2_O_2_. The concentration of ascorbic acid was adjusted to correspond to 1 mM of the monoanion (ascorbate) at all pH values to avoid reductant limitation (1.13 mM, 1.01 mM and 1.00 mM at pH 5.0, 6.0 and 7.0, respectively). Peroxygenase data represent the mean ± SD from independent replicates (*n* = 2), while the peroxidase data is based on a single measurement. TN = turnover number (i.e., the number of H_2_O_2_ molecules consumed per enzyme per second under the conditions used).

In line with previous observations for fungal AA9 LPMOs,^30,60^ the peroxygenase activity of the three enzymes also showed little pH-dependence (Figure 9). *Lp*LPMO10A^*Lp*^ maintained stable peroxygenase activity in the pH 5.0–7.0 range, while *Sm*LPMO10A and *Bl*LPMO10A displayed a slight increase and decrease with pH, respectively. Of note, the peroxygenase rates shown in Figure 9 were corrected for the rate of the peroxidase reaction (i.e., H_2_O_2_ depletion in the absence of substrate). Since the peroxidase reaction is likely suppressed in the reaction with substrate, the reported peroxygenase rates are underestimates. Noncorrected peroxygenase rates are shown in Figure S5. While the pH profiles in Figure S5B look slightly different from those shown in Figure 9, especially for *Bl*LPMO10A, the overall trends remain the same: there is little pH dependence, and this dependence is similar for all tested LPMOs. The similar pH-activity profiles of the three AA10 LPMOs show that *Lp*LPMO10A has not evolved features that make this enzyme particularly suitable for acting at low pH, despite the acidification that is associated with growth of *L. plantarum*.

The low pH-dependence of the activity of AA10 LPMOs in the pH 5–7 range aligns with the observation that the p*K*_a_′s of the copper coordinating histidine are lower than 4^44^ and the notion that the only titratable group in the second copper coordination sphere, a glutamate, likely also has a low p*K*_a_. Thus, all these residues stay deprotonated in the pH 5–7 region. Such lack of pH-dependence is also observed for AA9 LPMOs, despite the fact that in these LPMOs the only titratable group in the second copper coordination sphere is a histidine, with potentially a p*K*_a_ near pH 6 (see Kuusk et al., 2024 for an extensive discussion of this issue^60^).

Since *L. plantarum* will encounter pH values below 5, further studies of the activity of *Lp*LPMO10A at acidic pH would be of interest. Such studies would need to take the effect of pH on the reductant into account.^27^ In a preliminary experiment, using ascorbic acid-driven reactions, we observed that *Lp*LPMO10A retains full activity at pH 4.5, while activity at pH 4.0 is strongly reduced (Figure S6).

## Concluding Remarks

In this study, we present the functional characterization of *Lp*LPMO10A, a chitin-active LPMO from the nonpathogenic, chitinase-deficient bacterium *L. plantarum*. *Lp*LPMO10A expressed in both *L. plantarum* and *E. coli* displayed comparable catalytic activity and no host-specific post-translational modifications could be detected. Of note, post-translational modifications have been detected in the LPMO domains of a multidomain chitin-active LPMOs involved in virulence of *Pseudomonas aeruginosa*.^15^ The native *L. plantarum*-derived enzyme was used in most analyses to ensure biological relevance.

Analysis of H_2_O_2_-driven catalysis revealed that both *Lp*LPMO10A and *Sm*LPMO10A efficiently oxidize chitin, with little enzyme inactivation even in the presence of high amounts of H_2_O_2_. In general, the two enzymes seem very similar in almost all aspects, including a higher folding stability at pH 5.0 compared to pH 8.0, the composition of the hole hopping route in the protein core, redox stability during on- and off-pathway turnover, and the pH-dependence of on- and off-pathway reactions. The only major differences between the two enzymes concern the 2–3-fold higher initial rate in the peroxygenase reaction with chitin (at 100 μM H_2_O_2_) of *Sm*LPMO10A at each pH tested (Figure 9B) and the pH-dependence of substrate-binding. As to the latter, *Lp*LPMO10A binds the substrate less well than *Sm*LPMO10A and has a relative preference for lower pH compared to *Sm*LPMO10A. The similarity between the two enzymes is remarkable. Whereas *Sm*LPMO10A plays a clear role in chitin degradation by *S. marcescens*,^61,62^ *L. plantarum* does not grow on chitin (unpublished results).

Next to revealing the properties of *Lp*LPMO10A, the present study shines light on functional variation among closely related LPMOs. Our assessment of pH-dependence, the first of its kind for bacterial LPMOs, shows that, like for fungal AA9-type LPMOs, the reactivity of bacterial AA10 LPMOs hardly depends on pH in the 5–7 range, which is interesting considering the presence of different titratable groups in the second coordination spheres of the copper, as discussed above. At the same time, our comparison of multiple single domain chitin-active AA10-type LPMOs in subclade D of the phylogenetic tree, shows conspicuous functional differences. The most prominent difference is the much lower stability of *Bl*LPMO10A during chitin turnover. This lower stability is likely associated with a decreased ability to control the fate of radicals formed in the catalytic center, which could be due to β-chitin not being an optimal substrate and/or to a reduced ability to stabilize or dissipate radicals in a hydrophobic core that is less suited to do so than the hydrophobic cores of *Lp*LPMO10A and *Sm*LPMO10A (Figure 8). The lower ability of *Bl*LPMO10A to degrade chitin is remarkable since *B. licheniformis* produces multiple chitinases and can likely degrade chitin.

It has to be noted that the chitin used in laboratory experiments differs from natural substrates, in which the chitin may have another form, and may be part of complex copolymeric structures. It is conceivable that different chitin-active LPMOs have evolved to act on distinct chitin types or GlcNAc-rich materials and, as such, the differences and similarities revealed in our studies with β-chitin may not all be biologically relevant. For example, while *Bl*LPMO10A was the least efficient of the LPMOs when acting on β-chitin, its activity could differ when tested on other chitin-containing substrates, such as insect shell debris. In this respect, it is worth noting that almost all characterized bacterial LPMOs with no obvious role in chitin degradation are active on chitin;^15,63^ whether chitin is the true substrate of these enzymes remains to be seen. As for *Lp*LPMO10A, we have tested a wide variety of nonchitin, mostly polysaccharide substrates, including various (hemi)­celluloses, starch, pectin and peptidoglycan, without detecting activity (results not shown).

It is interesting to note that *Lp*LPMO10A tolerates relatively high H_2_O_2_ concentrations in vitro (concentrations up to 200 μM were tested; Figure 6). Several *L. plantarum* isolates can withstand considerable H_2_O_2_ concentrations, amounting to as high as 4–5 mM under laboratory conditions.^64,65^ It may be advantageous for the bacterium that *Lp*LPMO10A has the capacity to remain catalytically competent at elevated H_2_O_2_ levels. Interestingly, a study with *L. plantarum* CAUH2 showed an increase in expression of its LPMO under oxidative stress.^65^ This could indicate that LPMO production is adapted to the availability of the cosubstrate or perhaps even that the LPMO reaction is used by the bacterium to relieve oxidative stress.

Despite originating from a bacterium lacking a canonical chitinolytic system, *Lp*LPMO10A retains key structural and functional features of chitin-active AA10-type LPMOs. The conservation of these features, together with considerable stability under oxidative conditions, suggest that *Lp*LPMO10A may confer *L. plantarum* a selective advantage when encountering exogenous GlcNAc-containing materials, such as fungal debris or insect-derived chitin fragments. Perhaps, *Lp*LPMO10A contributes to ecological fitness by allowing feeding on chitinous materials that do not require canonical chitinases to be assimilated by the bacterium. Recent studies with *Lysobacter enzymogenes* link a chitin-active LPMO to antifungal activity and antagonism,^66^ providing another possible function for *Lp*LPMO10A. This potential role is particularly interesting in light of *L. plantarum* *’s* known ability to produce a broad range of antimicrobial compounds, including organic acids, H_2_O_2_, diacetyl, and bacteriocins, that contribute to antagonism against diverse pathogens.^67^ Perhaps, somehow, the LPMO is another of these antimicrobial compounds. Totally different roles, for example in cell wall remodeling, should also be considered. Overall, our study positions *Lp*LPMO10A as a functionally conserved but contextually atypical LPMO, highlighting the broader and still poorly understood biological roles of AA10-type LPMOs. Further studies, for example studies exploring possible natural substrates and physiological studies with knockout strains, are needed to fully understand the biological significance of *Lp*LPMO10A and related enigmatic LPMOs in microbial life.

## Materials and Methods

### Bioinformatic Analysis

To construct the phylogenetic tree, 47 AA10 sequences representing only the catalytic domains were retrieved from the CAZy database using accession numbers corresponding to biochemically and/or structurally characterized enzymes. Signal peptides and additional domains were manually removed prior to the generation of a multiple sequence alignment (MSA). The MSA was generated using the Clustal Omega tool, available through the EMBL-EBI online platform. The resulting alignment was used to generate a phylogenetic tree, which was visualized and annotated using the Interactive Tree of Life (iTOL) platform.^68^ Clade assignments were made based on previous classifications by Book et al.^69^ and Votvik et al.^70^ For organisms encoding LPMOs clustering with known chitin-active enzymes, the number of AA10 and GH18 genes present in the genome was determined using CAZy. This information was only available for bacterial genomes and not for those of viral or plant origin.

To compare the overall structures, substrate-binding surfaces, and putative hole-hopping pathways of the studied enzymes, AlphaFold3-predicted structures were generated for *Bl*LPMO10A and *Lp*LPMO10A, while the crystal structure of *Sm*LPMO10A (PDB ID: 2BEM^71^) was used as a reference. Although an NMR structure exists for *Bl*LPMO10A,^39^ its conformational flexibility makes direct comparison challenging; therefore, the AlphaFold3 model was used. Residues involved in substrate binding were identified based on an experiment-supported computational study of *Sm*LPMO10A in complex with chitin,^43,44^ while identification of residues potentially involved in hole hopping was guided by recent mechanistic insights into electron transfer pathways in *Sm*LPMO10A.^45,58^

### Cloning and Expression of *Lp*LPMO10A in *E. coli*

All LPMOs were expressed in *E. coli* using the pRSET expression vector (Invitrogen, Carlsbad, CA). For expression of *Lp*LPMO10A in *E. coli* (*Lp*LPMO10A^*Ec*^), the AA10 of *L. plantarum* WCFS1 without its signal peptide (residues 33–201; UniProt ID: F9UP60) was amplified from genomic DNA using PCR with primers containing overhangs corresponding to the pRSET B vector (forward primer: 5′-CGCAACAGGCGAATGCCCATGGCTTTGTGACGAACCC-3′; reverse primer: 5′-CAGCCGGATCAAGCTTTTACTGTACGTCAATATCTGAGACTTGAT-3′). The resulting PCR fragment was inserted into BsmI- and *Hin*dIII-digested pRSET vector using the In-Fusion HD Cloning Kit (Takara Bio/Clontech, Mountain View, CA), following the manufacturer’s instructions. The digested vector backbone contained the native signal peptide from the *S. marcescens* LPMO10A (*Sm*LPMO10A; residues 1–27), previously shown to enable efficient translocation of LPMOs to the periplasmic space in *E. coli*.^49,71^

The resulting plasmid was sequence-verified before transformation into chemically *competent* *E. coli* BL21 (DE3) Star cells (Invitrogen, Carlsbad, CA). Transformed cells were grown overnight in LB medium supplemented with 100 μg/mL ampicillin at 37 °C, 200 rpm. The overnight culture (10 mL) was used to inoculate 0.5 L Terrific Broth medium supplemented with 100 μg/mL ampicillin and 60 μL Antifoam 204 (Sigma-Aldrich, St. Louis, MO). Cultures were grown at 37 °C in a LEX-24 bioreactor (Harbinger Biotechnology and Engineering Corp., Markham, ON, Canada) without induction, due to the leakiness of the T7 promoter. After 24 h, cells were harvested by centrifugation, and periplasmic extracts were obtained using the osmotic shock method as previously described.^72^ Prior to enzyme purification, periplasmatic extract fractions were sterilized by filtration (0.2 μm) and stored at 4 °C.

### Cloning and Expression of *Lp*LPMO10A in *L. plantarum* WCFS1

Expression of *Lp*LPMO10A in *L. plantarum* WCFS1 (*Lp*LPMO10A^*Lp*^) was carried out using the pSIP expression system.^46,48^ The *Lp*LPMO10A gene (*lp_1697*), including the native signal peptide (residues 1–201), was amplified from genomic DNA using PCR with In-Fusion primers (forward: 5′-GGAGTATGATTCATATGTTGAGCACCAAGAAGAATCATGTAT-3′; reverse: 5′-CTGTAATTTGAAGCTTTTACTGTACGTCAATATCTGAGACTTGA-3′). The PCR fragment was cloned into NdeI- and *Hin*dIII-digested pSIP403 vector using the In-Fusion HD Cloning Kit (Takara Bio/Clontech, Mountain View, CA) according to the manufacturer’s instructions. The resulting plasmid was first transformed into One Shot TOP10 chemically competent *E. coli* cells (Invitrogen, Carlsbad, CA) and transformants were grown in Brain Heart Infusion (BHI) medium supplemented with 200 μg/mL erythromycin. The plasmid (pSIP_*Lp*LPMO10A) was sequence-verified by Eurofins Genomics (Ebersberg, Germany) before transformation into electrocompetent *L. plantarum* WCFS1, as previously described.^46,48^

For overexpression of *Lp*LPMO10A^*Lp*^, an overnight culture of *L. plantarum* WCFS1 harboring pSIP_*Lp*LPMO10A was used to inoculate 1 L of MRS medium (without Tween). Cultures were incubated statically at 37 °C until the optical density at 600 nm (OD_600_) reached ∼0.3, at which point expression was induced by addition of 10 ng/mL (final concentration) of the peptide pheromone SppIP (Caslo ApS, Lyngby, Denmark). Approximately 18 h postinduction, cells were removed by centrifugation at 5,000×*g* for 15 min at 4 °C, and the supernatant was collected.

The supernatant was concentrated to ∼100 mL using a Vivaflow 200 ultrafiltration system equipped with a 10 kDa PES membrane (Sartorius, Göttingen, Germany). Buffer exchange to 50 mM Tris-HCl (pH 9.0) was performed using five times the original supernatant volume (5 L) with the same Vivaflow system. The concentrated and buffer-exchanged sample was stored at 4 °C until subsequent purification.

### Purification of *Lp*LPMO10A

*Lp*LPMO10A produced in *L. plantarum* (*Lp*LPMO10A^*Lp*^) and *E. coli* (*Lp*LPMO10A^*Ec*^) was purified using a two-step protocol consisting of ion exchange chromatography followed by size-exclusion chromatography. For the periplasmic extract from *E. coli*, the sample was adjusted to buffer A (50 mM Tris-HCl, pH 9.0). The concentrated supernatant containing *Lp*LPMO10A^*Lp*^ was already in buffer A after buffer exchange of the culture supernatant. Protein samples were loaded onto a pre-equilibrated 5 mL HiTrap Q FF anion exchange chromatography column (Cytiva, Marlborough, MA) connected to an ÄKTA purifier FPLC system (Cytiva). *Lp*LPMO10A did not bind to the column and was collected in the flow-through fractions.

Flow-through fractions were pooled and concentrated using Amicon Ultra-15 centrifugal filters with a 10 kDa molecular weight cutoff (Merck Millipore, Burlington, MA). The concentrated protein was further purified by size-exclusion chromatography using a ProteoSEC Dynamaic 16/60 3–70 HR column (Protein Ark, Sheffield, U.K.), operated at 1 mL/min in 50 mM Tris-HCl (pH 8.0) containing 200 mM NaCl. Fractions containing the pure enzyme were pooled, concentrated, and the buffer was exchanged to 20 mM Tris-HCl (pH 8). Protein purity was assessed by SDS-PAGE, and protein concentrations were determined by measuring absorbance at 280 nm and applying the Beer–Lambert law using the theoretical extinction coefficient of mature *Lp*LPMO10A (46,410 M^–1^ cm^–1^).

### Other Enzymes

The LPMOs from *S. marcescens* (*Sm*LPMO10A) and *B. licheniformis* (*Bl*LPMO10A), and chitobiase from *S. marcescens* (*Sm*CHB) were expressed and purified as previously described.^62,73,74^

### Copper Saturation of LPMOs

Prior to use, all LPMOs were saturated with copper by incubating the enzymes, in 50 mM Tris-HCl (pH 8.0), on ice for 30 min with a 3-fold molar excess of Cu­(II)­SO_4_ (Merck, Darmstadt, Germany). After incubation, excess Cu­(II)­SO_4_ was removed using Amicon Ultra-15 centrifugal filters with a 10 kDa molecular weight cutoff (Merck Millipore, Burlington, MA). Copper was removed by stepwise dilution with 20 mL aliquots of 20 mM Tris-HCl, pH 8.0, until the theoretical free copper concentration was <2 nM. Copper-saturated enzymes were stored at 4 °C until further use.

### Whole Protein MALDI-ToF MS Analysis

Whole-protein MALDI-ToF mass spectrometry of *Lp*LPMO10A^*Lp*^ and *Lp*LPMO10A^*Ec*^ was performed using an UltrafleXtreme MALDI-TOF mass spectrometer (Bruker Daltonics GmbH, Bremen, Germany). A 0.5 μL aliquot of enzyme solution (in 20 mM NaOAc pH 5.6) was mixed with 2.5 μL matrix solution (20 mg/mL 2,5-dihydroxybenzoic acid and 1 mM NaCl in 30% v/v acetonitrile) on an MTP 384-ground steel target plate (Bruker Daltonics) leading to a protein concentration between 20 and 30 μM. Samples were air-dried, and spectra were collected and analyzed using FlexAnalysis software (Bruker, version 3.4).

### LPMO Activity on α- and β-Chitin

Unless otherwise stated, reactions were performed in triplicate using 1 μM LPMO and 10 g/L shrimp shell (*Pandalus borealis*) α-chitin (purchased from Chitinor AS; Senjahopen, Norway; milled to a particle size of ∼200 μm) or squid pen β-chitin (France Chitin, Orange, France, batch number 20140101; milled to a particle size of 75–200 μm) in 25–50 mM sodium acetate buffer (pH 5.0) or 50 mM Tris-HCl pH 8.0. Reactions were initiated by the addition of 1 mM ascorbic acid and incubated at 37 °C and 800 rpm. For reactions with exogenous H_2_O_2_ (0–200 μM), H_2_O_2_ was added prior to the addition of ascorbic acid. At selected time points, reactions were stopped by filtration through a 96-well filter plate (Millipore) using a Millipore vacuum manifold. Control reactions lacking substrate, ascorbic acid, and/or LPMO were included.

To quantify soluble oxidized chito-oligosaccharides produced by the LPMOs, filtrates were incubated overnight at 37 °C with 1 μM *Sm*CHB under static conditions. This treatment converted all soluble oxidized products into a mixture of native monomer (GlcNAc) and oxidized dimer (GlcNAc–GlcNAc1A).^74^ The monomer (GlcNAc) was quantified as a proxy for chitin conversion, whereas in some cases (Figure S2) also the oxidized dimer was quantified.

### Detection of Longer Oxidized Chitin-Derived Products – HILIC-UV

To generate a qualitative profile of the oxidized chito-oligosaccharides released during LPMO action on β-chitin, filtrates were analyzed using an Agilent 1290 Infinity II UHPLC system (Agilent, Santa Clara, CA) equipped with a 2.1 × 150 mm^2^, 130 Å, 1.7 μm BEH Amide column (Waters) and a 2.1 × 5 mm^2^, 130 Å, 1.7 μm BEH Amide VanGuard precolumn, as previously described.^74^ Products were detected by monitoring absorbance at 195 nm.

### Detection of Longer Oxidized Chitin-Derived Products – MALDI-ToF MS

Reaction products were analyzed qualitatively by matrix-assisted laser desorption/ionization time-of-flight mass spectrometry (MALDI-ToF MS) using an UltrafleXtreme system (Bruker Daltonics, Bremen, Germany) equipped with a 337 nm nitrogen laser. One μL of filtrate from the LPMO reactions was applied to an MTP 384 ground steel target plate TF (Bruker Daltonics) and mixed with 2 μL of 2,5-dihydroxybenzoic acid matrix solution (9 mg/mL in 30% acetonitrile). Samples were dried with a stream of air before analysis. Mass spectra were recorded and processed using FlexAnalysis software (Bruker, version 3.4).

### Quantification of Soluble Oxidized Chitin-Derived Products – Rezex-UV

Quantification of *N*-acetylglucosamine (GlcNAc) and oxidized chitobiose (GlcNAcGlcNAc1A; DP2ox) was performed using a Dionex RSLC system (Dionex, Sunnyvale, CA) equipped with a Rezex RFQ-Fast Acid H^+^ (8%) column (100 × 7.8 mm^2^; Phenomenex, Torrance, CA) operated at 85 °C. Eight μL of *Sm*CHB-treated samples were injected and separated isocratically using 5 mM sulfuric acid as the mobile phase at a flow rate of 1 mL/min with a total run time of 6 min. Products were detected by monitoring absorbance at 194 nm, and data were processed using the Chromeleon software 7.0.

Quantification was based on calibration curves generated with GlcNAc standards (31.25–1000 μM) and GlcNAcGlcNAc1A standards (25–800 μM). GlcNAc (95% purity) was obtained from Megazyme (Bray, Ireland), while oxidized dimer standards were prepared in-house by complete oxidation of *N*-acetyl-chitobiose (Megazyme, 95% purity) using a chitooligosaccharide oxidase from *Fusarium graminearum* (*Fg*ChitO^75^), as previously described.^74^

### Binding to β-Chitin

Binding of LPMOs to β-chitin in the presence of ascorbic acid was assessed using an adapted Bradford protocol for low protein concentrations.^45,76^ Binding reactions contained 10 g/L β-chitin (particle size 75–200 μm) and 2 μM LPMO in either 20 mM sodium acetate (pH 5.0) or 20 mM Tris-HCl (pH 8.0). Reactions were started by addition of 1 mM ascorbic acid and incubated at 22 °C with shaking at 800 rpm in an Eppendorf ThermoMixer C (Eppendorf, Hamburg, Germany). At selected time points (t = 0, 2.5, 5, 15, 30 min), samples were taken and rapidly subjected to vacuum filtration using a 96-well filter plate (0.45 μm) to separate unbound protein from protein bound to the β-chitin. The amount of bound protein was determined by measuring the concentration of unbound protein in the supernatant using the adapted Bradford assay.^76^ 100% unbound was determined using a control reaction lacking chitin.

### Thermal Stability of LPMOs Assessed by Differential Scanning Fluorimetry

The apparent melting temperature (T_*m*,*app*_) of the LPMOs was determined using differential scanning fluorimetry (DSF) and SYPRO Orange (Thermo Fisher Scientific, Waltham, MA), a dye that increases in fluorescence upon binding to hydrophobic regions exposed during protein unfolding.^77^ To investigate the effect of pH, a universal buffer (final concentration 10 mM K_3_BO_3_, CH_3_COONa, Na_2_HPO_4_) was prepared at pH 3–7. Additionally, 25 mM sodium acetate (pH 5.0) and 25 mM Tris-HCl (pH 8.0) were used. To ensure complete copper saturation of the LPMOs (0.1 mg/mL final concentration), a 5-fold molar excess of Cu­(II)­SO_4_ was added to the reaction mixture 5 min before the addition of 1× SYPRO Orange and the start of the unfolding experiment.

Thermal unfolding was monitored by measuring fluorescence (Ex/Em = 490/530 nm) in a StepOnePlus Real-Time PCR instrument (Thermo Fisher Scientific), gradually increasing the temperature from 25 to 99 °C over 50 min. The apparent T_*m*_ was determined from the maximum of the negative derivative of the fluorescence signal (−dF/dT). All measurements were performed in quadruplicates (*n* = 4).

### Peroxidase Reactions with 2,6-Dimethoxyphenol

The peroxidase activity of the LPMOs was assessed using the method described by Breslmayr et al.^53^ Reaction mixtures contained 2 μM LPMO, 2 mM 2,6-dimethoxyphenol (2,6-DMP), and 200 μM H_2_O_2_ in 50 mM sodium acetate buffer (pH 5.0). Assays were performed at 30 °C in a 96-well plate with a final reaction volume of 100 μL. Formation of the product was monitored at 473 nm and recorded every 5 s for 10 min using a Varioskan LUX plate reader (Thermo Fisher Scientific, Waltham, MA).

All reactions were performed in triplicate, and control reactions lacking enzyme were included. The initial velocity (ΔA_473_/min) was determined from the linear part of the progress curve using linear regression.

### Oxidase Activity Assay with HRP/Amplex Red

The H_2_O_2_ production assays were performed according to the approach previously described by Kittl et al.^54^ Reactions were performed in 25 mM sodium acetate buffer (pH 5.0) or 25 mM Tris-HCl (pH 8.0) at 30 °C. Reaction mixtures contained 3 μM LPMO or Cu­(II)­SO_4_, 5 U/mL horseradish peroxidase (HRP), and 100 μM Amplex Red. Mixtures were preincubated for 3 min at 30 °C, and the reaction was initiated by adding ascorbic acid to a final concentration of 1 mM. The final reaction volume was 100 μL. Hydrogen peroxide formation was quantified by monitoring the HRP-catalyzed conversion of Amplex Red to resorufin. The increase in absorbance at 563 nm was measured every 20 s for 2 h using a Varioskan LUX plate reader (Thermo Fisher Scientific, Waltham, MA). H_2_O_2_ production was quantified using a standard curve (0–20 μM H_2_O_2_) prepared in the same buffer that contained 1 mM ascorbic acid.

All reactions were performed in triplicate, including control reactions lacking LPMO and/or containing 3 μM Cu­(II)­SO_4_. Apparent H_2_O_2_ production rates (nM/s) were derived from the initial linear part of the progress curves using linear regression.

### Electrochemical Monitoring of H_2_O_2_ Consumption

The peroxidase and peroxygenase activities of the LPMOs were assessed by real-time electrochemical monitoring of H_2_O_2_ consumption as originally described by Schwaiger et al.^30^ Briefly, [H_2_O_2_] was detected amperometrically using an Autolab PGSTAT101 potentiostat (Metrohm, Herisau, Switzerland) connected to a gold rotating disk electrode coated in a layer of Prussian blue and protected by a layer of Nafion (Sigma-Aldrich, St. Louis, MO). Measurements were performed in a 4 mL volume at 37 °C using an Ag|AgCl electrode in 3 M KCl as the reference electrode and *a* platinum sheet as the counter electrode. Time-resolved data were acquired using NOVA 1.1 software from Metrohm and processed to correct for system drift. The current was converted to [H_2_O_2_] using a standard curve recorded at the beginning of each run.^30^ All reactions were performed in 50 mM NaOAc (pH 5.0) or MES (pH 6.0 or 7.0) buffer, and 100 mM KCl as the electrolyte. Five μM EDTA, a concentration too low to deplete Cu from LPMOs, was included in all runs to scavenge free metal ions present in the reaction solution or released from inactivated LPMOs.

Peroxidase activity was measured in the absence of chitin. Reactions contained 0, 1, 1.5, or 2 μM LPMO and 100 μM H_2_O_2_ and were started by the addition of 1 mM ascorbic acid. The linear initial rate of H_2_O_2_ consumption was estimated using a straight-line fit of the first 4 s following addition of ascorbic acid. The concentration of H_2_O_2_ turned over in each reaction before enzyme inactivation was estimated using a linear fit of the tail of each run, once the rate of hydrogen peroxide consumption became linear and identical to the rate in a control reaction without enzyme, reflective of enzyme inactivation. The y-intercept of this linear fit subtracted from the starting [H_2_O_2_] provides an estimate of the concentration of H_2_O_2_ consumed enzymatically prior to full enzyme inactivation, Δ­[H_2_O_2_]_max_. The ratio between Δ­[H_2_O_2_]_max_ and [LPMO], obtained by linear regression, defines *n*_max_, the average number of peroxidase turnovers performed by one LPMO before inactivation. These experiments were inspired by Kuusk et al.,^24^ who measured ascorbate-peroxidase activity by monitoring ascorbic acid depletion.

For measurement of the peroxygenase activity LPMOs were incubated with chitin for at least 1 h at 22 °C with agitation prior to measurement. The LPMO and chitin mixture was then added to the temperature-controlled reaction chamber, H_2_O_2_ was added stepwise to 100 μM final concentration to calibrate the sensor,^30^ then reactions were initiated by addition of ascorbic acid. The observed initial rate of H_2_O_2_ consumption (*v*_obs_) was estimated by a straight-line fit of the first 4 s. Residual activity after consumption of the first 100 μM of H_2_O_2_ was estimated from the difference between the initial *v*_obs_ and the *v*_obs_ obtained upon addition of another 100 μM of H_2_O_2_. Conservative estimates of the peroxygenase rate were made by subtracting the linear background rate of H_2_O_2_ consumption with chitin but without LPMO (*v*_bg_) and the initial rate of H_2_O_2_ consumption in the absence of chitin (*v*_off‑path_) from *v*_obs_ to give the corrected initial rate of the peroxygenase activity (*v*_enz_). The per-second turnover number (TN) of peroxygenase activity was calculated from *v*_enz_ by dividing by the [LPMO].^30^

$$\mathrm{TN}=\frac{v_{\mathrm{enz}}}{\left[\mathrm{LPMO}\right]}=\frac{v_{\mathrm{obs}}-v_{\mathrm{off}-\mathrm{pat}h}-v_{\mathrm{bg}}}{\left[\mathrm{LPMO}\right]}$$

## Supplementary Material



## Notes

Structural comparison of *Bl*LPMO10A, *Lp*LPMO10A, and *Sm*LPMO10A; comparison of α- and β-chitin degradation; identification of β-chitin oxidation products; chitin degradation assays ± H_2_O_2_; pH dependence of H_2_O_2_ consumption rates in reactions ± LPMO and ± β-chitin; and activity of *Lp*LPMO10A at acidic pH (PDF)
