# Supplementary material for: Homologous Expression and Characterization of an LPMO from Commensal Lactiplantibacillus plantarum WCFS1 and Comparison with Related Enzymes from Chitin-Degrading Bacteria
Source: ACS Omega. 2026 Jun 17;11(25):37755–70. doi: 10.1021/acsomega.6c02723 (PMC13325158; doi:10.1021/acsomega.6c02723)
Supplement: Supplementary file 1 [file ao6c02723_si_001.pdf]

Supplementary information for

Homologous expression and characterization of an LPMO from commensal *Lactiplantibacillus plantarum* WCFS1 and comparison with related enzymes from chitin-degrading bacteria

Hanne Berggreen[1], Tom Z. Emrich-Mills[1], Geir Mathiesen[1], Vincent G. H. Eijsink[1*] and Zarah Forsberg[1*]

[1] Faculty of Chemistry, Biotechnology and Food Science, The Norwegian University of Life Sciences (NMBU), Norway

*To whom correspondence should be addressed: vincent.eijsink@nmbu.no and zarah.forsberg@nmbu.no

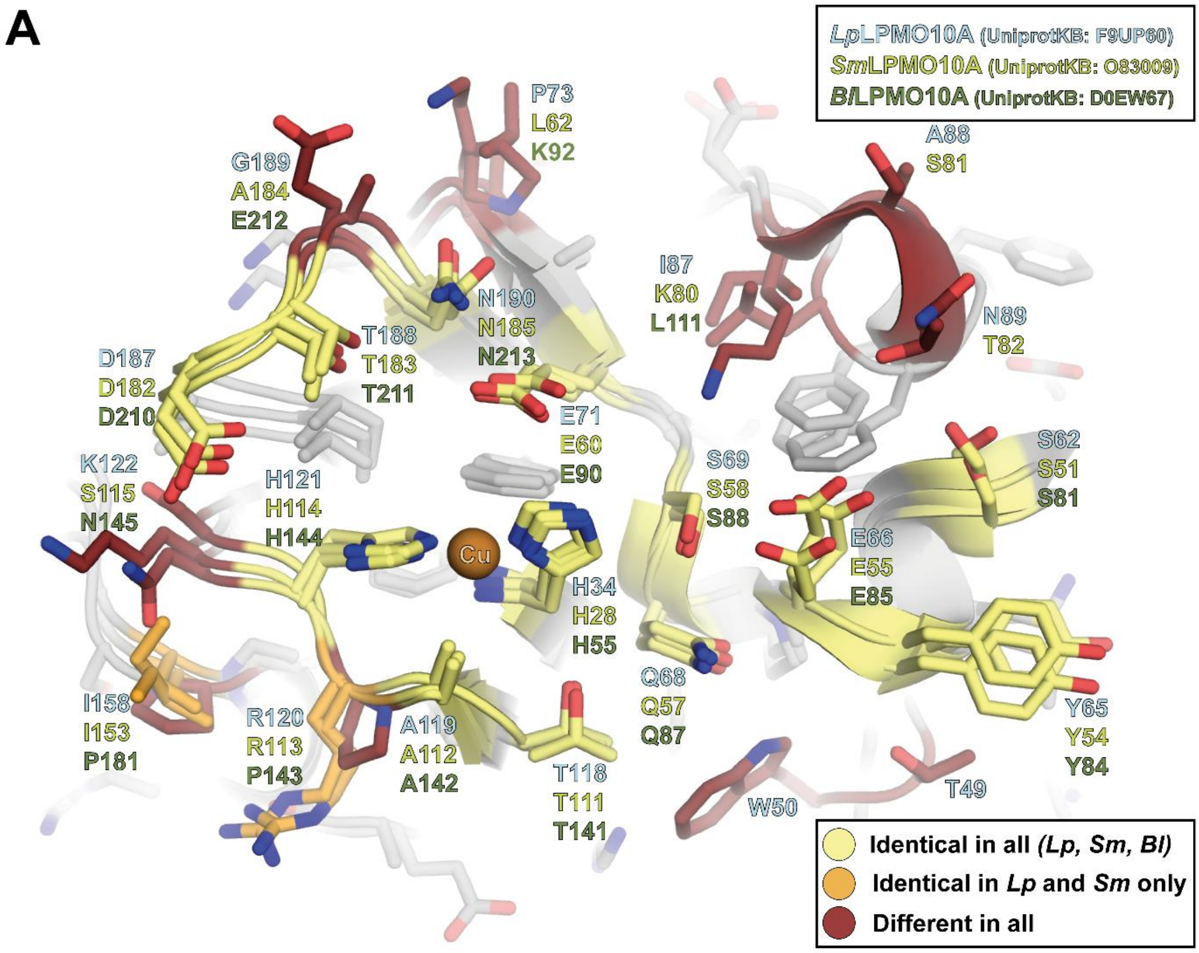

B

```
Lp-34  HGFVTNPGGRAYLGSTWYPGGPLNTNIGSVMYEPQSIEA----PQNTFIDGKIASAGIAN 89
Sm-28  HGYVESPASRAYQCKLQ-----LNTQCGSVQYEPQSVEGLKGFPQAGPADGHIASADKST 82
Bl-55  HGFIEKPGSRAALCSEAF--GFLNLNCGSVMYEPQSLEAKKGFPHSGPADGQIASAGGLF 112
       **:: .*..**   .       ** : *** *****:*.    *:    **:****.

Lp-90  FAPLDEQNAKRWYKTPVKAGNLSVTWQLTARHKTSTWDYYITKPSWNPNAPLKFSDFKKI 149
Sm-83  FFELDQQTPTRWNKLNLKTGPNSFTWKLTARHSTTSWRYFITKPNWDASQPLTRASFDLT 142
Bl-113 GGILDQQSENRWFKHIMTGGEHTFTWTYTAPHNTSQWHYYITKKGWDPDKPLKRADFELI 172
          **:*. .** *  :. *  :.**  ** *.*: * *:*** .*: . **. :.*.

Lp-150 A--SYNDNGAIPSEFVTHQVNISANEKGYQVLLSVWNIADTGNAFYQVSDIDVQ--  201
Sm-143 PFCQFNDGGAIPAAQVTHQCNIPADRSGSHVILAVWDIADTANAFYQAIDVNLSK-  197
Bl-173 GA--VPHDGSPASRNLSHHIYIPEDRLGYHVILAVWDVADTENAFYQVIDVDLVNK  226
             ..*:  :  ::*:  *  :. * :*:*:**::*** *****. *:::
```

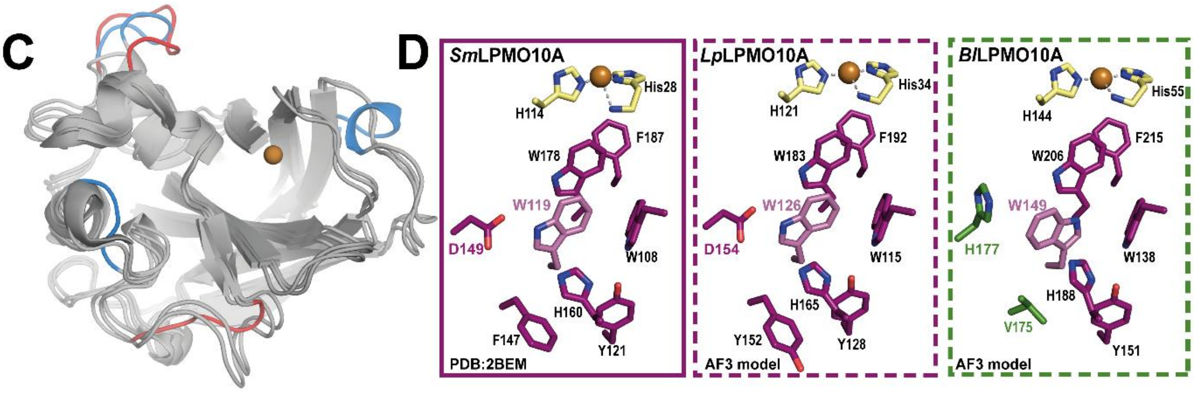

Figure S1. Structural comparison of *Lp*LPMO10A, *Sm*LPMO10A, and *Bl*LPMO10A. Panel A shows a superposition of the three structures: *Sm*LPMO10A (PDB ID: 2BEM) and AlphaFold3 models of *Lp*LPMO10A and *Bl*LPMO10A, shown as a top view of the substrate-binding surface with the centrally positioned copper active site. Residue conservation is color-coded; yellow – fully conserved in all three enzymes; orange – conserved between *Lp*LPMO10A and *Sm*LPMO10A only (Arg120 and Ile158, *Lp* numbering); red – different in all three enzymes. Non-labeled side chains with grey carbons belong to buried (non–surface-exposed) residues. Previous studies have suggested that the poorly conserved outer residues (orange and red) do not play a significant role in substrate binding[1]. Panel B shows a Clustal Omega sequence alignment highlighting substrate-binding residues shown in panel A (yellow); disulfide-forming cysteines (green) and residues discussed in relation to hole hopping (purple)[2]. Panel C illustrates a structural overlay showing the rather minimal predicted variation in loop conformation between the three enzymes (*Bl* in blue and *Lp* in red). Panel D shows a comparison of the main hole-hopping pathways. Previous studies have shown that the variation at position 149/154/177 (Asp149/ Asp154 in *Sm*/*Lp*, His177 in *Bl*) and the related change in the confirmation of the nearby Trp residue (119/126/149) affect H_2O_2 tolerance[3].

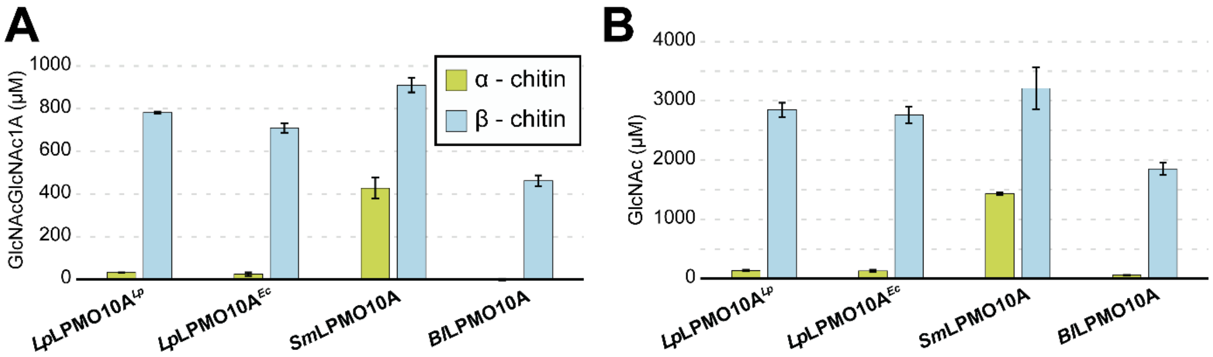

Figure S2. Comparison of LPMO activity on α- and β-chitin. All LPMOs were incubated with either α- or β-chitin (10 g/L) in reactions containing 1 µM copper-saturated LPMO and 1 mM ascorbic acid in 50 mM Tris-HCl (pH 8.0). Reactions were incubated for 24 h at 37 °C with shaking at 800 rpm. After incubation, samples were filtered, and the soluble fractions were treated with *Sm*CHB (chitobiase) to convert longer oxidized chito-oligosaccharides into DP2ox (GlcNAcGlcNAc1A; panel A) and native monomer (GlcNAc; panel B) for quantification. All reactions were performed in triplicate, and error bars represent the standard deviation (n=3).

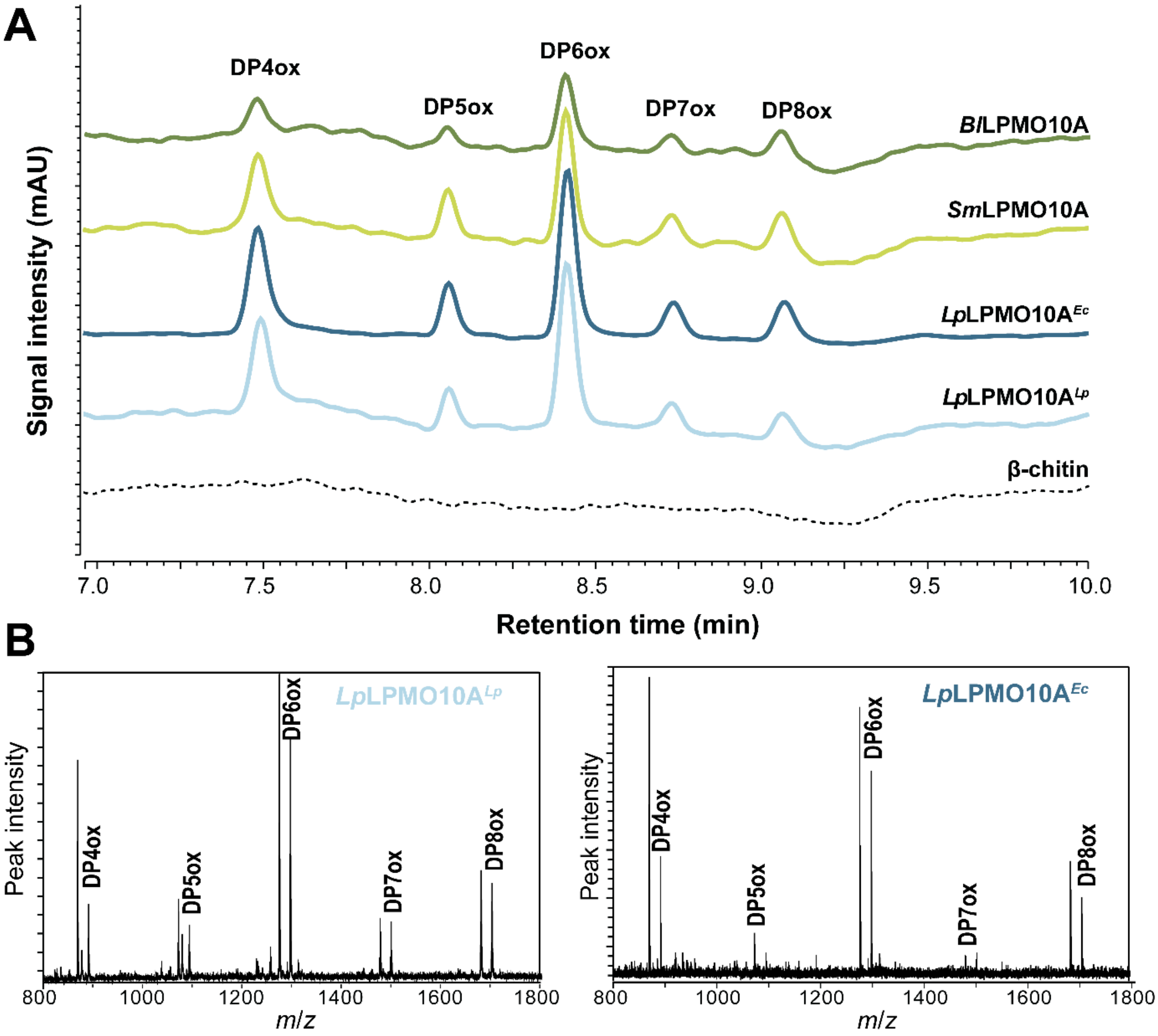

Figure S3. Analysis of oxidized chito-oligosaccharides generated by LPMOs acting on β-chitin. Panel A shows chromatographic analysis of soluble oxidized chito-oligosaccharides released by the four LPMOs. Reaction mixtures contained 10 g/L β-chitin, 1 μM copper-saturated LPMO, in 50 mM Tris-HCl (pH 8.0), and with 1 mM ascorbic acid, and were incubated at 37 °C with shaking at 800 rpm for 18 h. Filtered samples were analyzed by HILIC–UV chromatography. Colored solid lines show peaks corresponding to LPMO-generated oxidized chito-oligosaccharides (DP4ox to DP8ox) produced by *Bl*LPMO10A, *Sm*LPMO10A, *Lp*LPMO10A^{Ec}, or *Lp*LPMO10A^{Lp}. The dotted black line represents a control reaction without LPMO, showing the chitin background. Product formation was not observed in additional control reactions lacking ascorbic acid. The minor peaks appearing between the major DPox peaks (for *Bl*LPMO10A and *Sm*LPMO10A) may be attributed to partially deacetylated oxidized chito-oligosaccharides. Due to the high degree of acetylation of β-chitin, these species are present at low abundance. Peaks eluting before 7.0 min originate from buffer components and were omitted from the figure. Panel B shows MALDI-ToF MS analysis of oxidized chito-oligosaccharides (DP4–DP8) generated by *Lp*LPMO10A upon incubation with β-chitin. Reactions contained 1 μM *Lp*LPMO10A and 10 g/L β-chitin in 20 mM Tris-HCl (pH 8.0) supplemented with 1 mM ascorbic acid and were incubated at 37 °C and 800 rpm for 24 h. Filtered reaction mixtures were analyzed by MALDI-ToF MS. The left spectrum corresponds to *Lp*LPMO10A^{Lp} and the right spectrum to *Lp*LPMO10A^{Ec}. Each product gives multiple signals; for example, for DP6ox, the two main signals have *m/z* values of 1275 and 1297 representing the sodium adduct [DP6ox + Na]$^+$ and the sodium adduct of the sodium salt of oxidized chitohexose [DP6ox – H + 2 Na]$^+$ in its aldonic acid form, respectively.

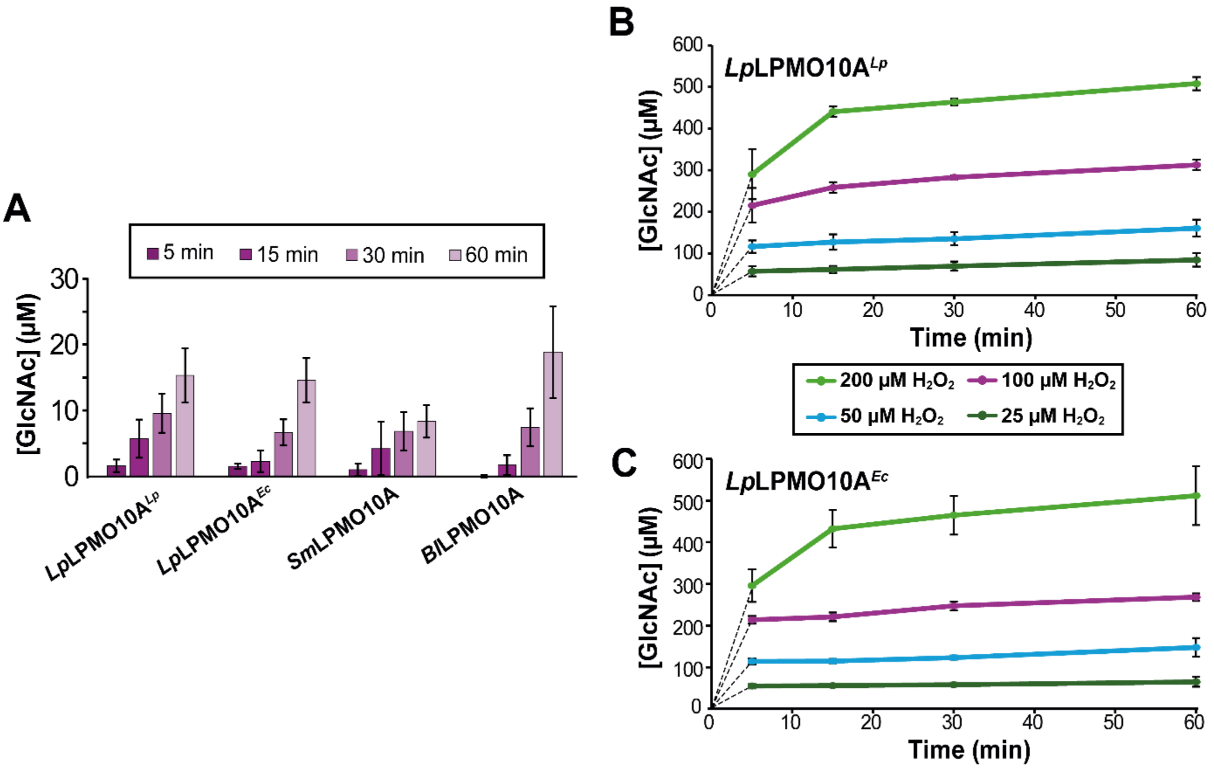

Figure S4. Degradation of β-chitin with and without supplemented H_2O_2. Panel A shows product formation for all four enzymes using the same conditions as in Figure 6 (1 µM LPMO, 10 g/L β-chitin, 1 mM AscA in 50 mM NaOAc pH 5.0) with the exception that no H_2O_2 was supplemented. Panel B and C show time-courses for chitin degradation by 1 µM *Lp*LPMO10A^{Lp} (B) and 1µM *Lp*LPMO10A^{Ec} (C) in the presence of different initial concentrations of exogenous H_2O_2 (0-200 µM) and ascorbic acid (1 mM) in 50 mM NaOAc (pH 5.0). Data for *Lp*LPMO10A^{Lp} are also shown in Figure 6A, and the experimental setup was identical to that described for Figure 6. Of note, the results shown in panel A cannot be directly compared to the results shown in Figure S2B because the two experiments were run at different pH and because pH has a strong effect on reductant-driven LPMO reactions[4].

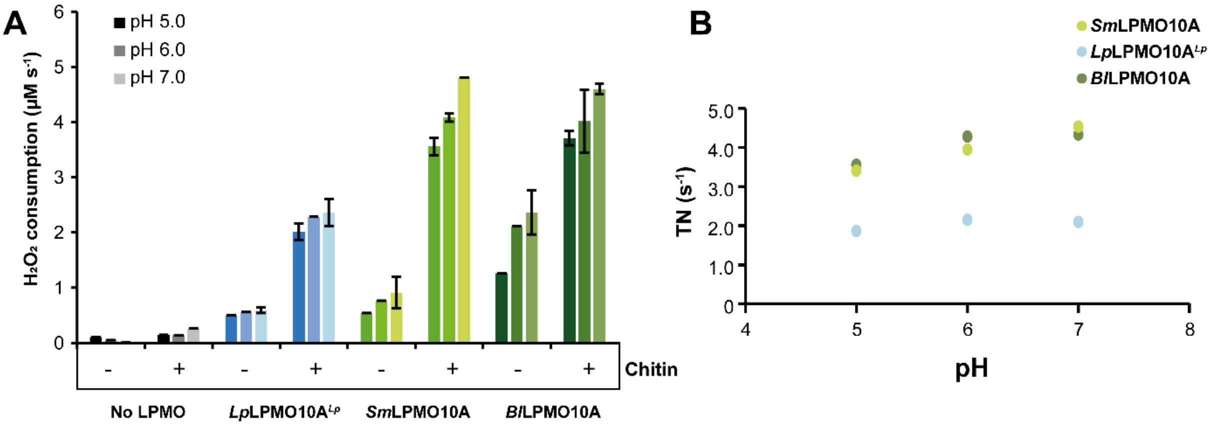

Figure S5. pH-dependence of the H_2O_2 consumption rate in reactions with and without LPMO and in the presence or absence of β-chitin. Data for panel A was gathered every 0.08 s and H_2O_2 consumption rates were derived from a linear fit of the first 3 seconds after addition of ascorbic acid, to a final concentration of 1 mM of the ascorbate monoanion (1.13 mM, 1.01 mM and 1.00 mM at pH 5.0, 6.0 and 7.0, respectively). Bars with errors are means ± standard deviation (n=2). Bars without error are single measurements. Experiments were conducted in 50 mM NaOAc, pH 5.0, or 50 mM MES buffer, at pH 6.0 or 7.0, at 37 °C using 1 µM LPMO and 100 µM H_2O_2. Panel B shows the pH dependence of the rate of the peroxygenase reaction without correction for the rate of the peroxidase reaction observed in the reaction without chitin.

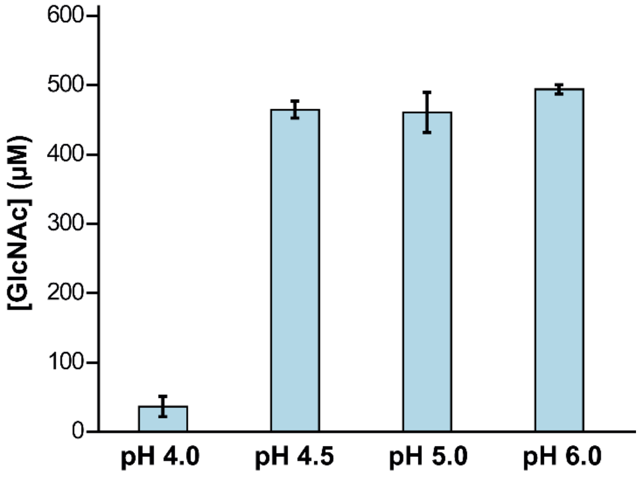

Figure S6. Activity of *Lp*LPMO10A at acidic pH. The reaction mixtures contained 10 g/L β-chitin, 1 µM LPMO, 50 mM sodium acetate pH 4.0, 4.5, 5.0 or 6.0, 200 µM H_2O_2 and 1 mM ascorbic acid and were incubated for 30 min at 37 °C, 800 rpm. Soluble reaction products were collected by filtration and subsequently degraded with *Sm*CHB, after which GlcNAc was quantified. Note that reduction likely becomes limiting at pH 4.0[5] and that the lack of product formation at this pH does not necessarily reflect a lack of catalytic competence of the enzyme.

Supplementary references

1. Bissaro, B.; Isaksen, I.; Vaaje-Kolstad, G.; Eijsink, V. G. H.; Røhr, Å. K., How a lytic polysaccharide monooxygenase binds crystalline chitin. *Biochemistry-Us* **2018,** *57* (12), 1893-1906.

2. Ayuso-Fernández, I.; Emrich-Mills, T. Z.; Haak, J.; Golten, O.; Hall, K. R.; Schwaiger, L.; Moe, T. S.; Stepnov, A. A.; Ludwig, R.; Cutsail III, G. E.; Sørlie, M.; Røhr, Å. K.; Eijsink, V. G. H., Mutational dissection of a hole hopping route in a lytic polysaccharide monooxygenase (LPMO). *Nat. Commun.* **2024,** *15* (1), 3975.

3. Ayuso-Fernández, I.; Emrich-Mills, T. Z.; Golten, O.; Forsberg, Z.; Hall, K. R.; Nagy, L. G.; Sørlie, M.; Kjendseth Røhr, Å.; Eijsink, V. G. H., Redox robustness drives LPMO evolution. *Proc. Natl. Acad. Sci. U.S.A.* **2026,** *123* (3), e2521617123.

4. Golten, O.; Ayuso-Fernández, I.; Hall, K. R.; Stepnov, A. A.; Sørlie, M.; Røhr, A. K.; Eijsink, V. G. H., Reductants fuel lytic polysaccharide monooxygenase activity in a pH-dependent manner. *FEBS Lett.* **2023,** *597* (10), 1363-1374.

5. Schwaiger, L.; Csarman, F.; Chang, H.; Golten, O.; Eijsink, V. G. H.; Ludwig, R., Electrochemical monitoring of heterogeneous peroxygenase reactions unravels LPMO kinetics. *ACS Catal.* **2024,** *14* (2), 1205-1219.
